# Antimicrobial Polymers in the Nano-World

**DOI:** 10.3390/nano7020048

**Published:** 2017-02-22

**Authors:** Marta Álvarez-Paino, Alexandra Muñoz-Bonilla, Marta Fernández-García

**Affiliations:** 1 Centre for Biomolecular Sciences, School of Pharmacy, University of Nottingham, Nottingham NG7 2RD, UK; 2 Instituto de Ciencia y Tecnología de Polímeros (ICTP-CSIC); C/ Juan de la Cierva 3, Madrid 28006, Spain

**Keywords:** polymers, antimicrobial, health, food, agriculture, water purification, textile

## Abstract

Infections are one of the main concerns of our era due to antibiotic-resistant infections and the increasing costs in the health-care sector. Within this context, antimicrobial polymers present a great alternative to combat these problems since their mechanisms of action differ from those of antibiotics. Therefore, the microorganisms’ resistance to these polymeric materials is avoided. Antimicrobial polymers are not only applied in the health-care sector, they are also used in many other areas. This review presents different strategies that combine nanoscience and nanotechnology in the polymer world to combat contaminations from bacteria, fungi or algae. It focuses on the most relevant areas of application of these materials, viz. health, food, agriculture, and textiles.

## 1. Introduction

The terms nanoscience and nanotechnology are widely used to label a variety of products. These terms are quite prevalent and appear in the news and advertisements in reference to new discoveries and exciting new products. Nowadays, the nano-world is everywhere. In the nanotechnology field, polymers are a major area of research; they have managed to greatly improve our society. Polymers possess highly desirable characteristics such as high strength or modulus to weight ratios (light weight but comparatively stiff and strong), toughness, resilience, resistance to corrosion and lack of conductivity (heat and electrical) among others, whilst being comparatively cheap. Many of these characteristics make them perfect candidates for their utilization in multiple applications; such as in biomedical devices, health care, food, agriculture, catalysis, electronics, environment, renewable energy or textiles [1].

In addition to the characteristics mentioned above, polymers can also show antimicrobial properties. Antimicrobial polymeric materials can be applied in the areas just stated and can avoid the resistance problems associated with antibiotics use. An antimicrobial agent can be defined as an agent that kills microorganisms or inhibits their growth. In general, the potency of such antimicrobial agents is directly proportional to their toxicity towards humans. For this reason, the development of potent but non-toxic antimicrobial polymers is much needed and pursued. In this sense, researchers look for the structural parameters that determine their activity, new structures or new mechanisms of action [2,3,4,5,6,7,8,9] to tune their potency and toxicity. Although there are numerous examples of antimicrobial polymeric materials, most of them are comprised of cationic polymeric systems with quaternary nitrogen groups; polymers mimicking natural peptides; halogen polymers; chitosan derivatives; and silver- and titania-nanocomposites.

## 2. Areas of Application

In this article, we review research carried out over the past decade on antimicrobial polymers and in particular, focusing on nanopolymeric materials [10,11]. The application of these materials can be categorized based on their main areas of application as follows: (i) health care and biomedical devices; (ii) food-related activities; (iii) environmental science and (iv) textile industry.

### 2.1. Health Care and Biomedical Devices

The main objectives of polymer researchers when developing novel materials for health care-related purposes, are to lower the risk of diseases transmission and spread of infections and to avoid biofilm formation. The search for new more efficient antibiotics is continuously increasing. However, and as mentioned above, antibiotic-resistant bacteria are becoming a great threat to human health mainly due to the abuse and the improper use of antibiotics. In this section, we present the health-care related topics where antimicrobial effect is of essence.

#### 2.1.1. Drug Delivery

Antibiotics administration via traditional routes such as intravenous, oral, ocular, pulmonary delivery, is challenging due to its systemic nature and lack of control over release rates. Hence, large and multiple doses are necessary to maintain therapeutic concentrations at the infection site. This, may lead to undesirable side-effects and toxicity. Besides, many antibiotics present low aqueous solubility and stability, limited bioavailability and low compliance. Thus, it is necessary to overcome these problems to reduce the undesirable effects of using excessive antimicrobial drugs.

Polymers, in particular polymeric nanoparticles (NPs; particles with dimensions in the nanosize range), are a promising alternative for controlled delivery and release of antibiotics, raising the drug’s effectiveness [12]. NPs can be colloidal in nature, biodegradable, biocompatible and similar in behaviour to intracellular pathogens. These colloidal carriers can be rapidly taken up by the microorganisms, with subsequent intracellular release of the drug. Both synthetic and naturally occurring materials have been assessed for their potential for drug delivery. Biodegradable spheres prepared from poly(lactide) (PLA) and its copolymers with glycolide, poly(lactic-*co*-glycolic acid) (PLGA), can encapsulate drugs and release them in a controlled way, depending on the method of microencapsulation and the physico-chemical properties of the polymer and the drug. Natural polyelectrolytes such as gellan gum (Gg), pectin hyaluronic acid (Hy), dextran and chitosan (Ch) are also attractive for medical applications due to their biocompatible and biodegradable nature. For example, rifampicin antibiotic (RIF)-loaded PLGA NPs have been prepared by the oil-in-water emulsification-solvent diffusion method. The effect of the nanoencapsulation on the antibacterial activity of RIF was evaluated against Gram-positive bacteria including *Staphylococcus aureus* (ATCC 6538P), methicillin-resistant *S. aureus* (MRSA) (clinical isolate) and *Bacillus subtilis* (ATCC 6633), and two Gram-negative bacteria *Pseudomonas aeruginosa* (ATCC 9027) and *Escherichia coli* (ATCC 8739). RIF-PLGA NPs were found to considerably improve the antibacterial efficacy of the drug against the three Gram-positive bacteria as showed by the well diffusion method [13].

In tuberculosis, the second most deadly infectious disease, *Mycobacterium tuberculosis* evades clearance mechanisms within macrophages through suppression of intracellular reactive oxygen and nitrogen species and pro-inflammatory cytokines. Dube et al. [14] proposed developing NPs functionalized with ligands able to modulate the cellular immune response and concurrently deliver the drug. They designed NPs consisting of a 1,3-β-glucan- functionalized chitosan shell and a PLGA core to stimulate the production of intracellular reactive oxygen and nitrogen species, and the secretion of pro-inflammatory cytokines such as interleukin-12 (IL-12), tumour necrosis factor-α (TNF-α) and interferon-gamma (IFN-γ). In addition, these NPs were loaded with rifampicin to promote its delivery inside human alveolar-like macrophages (ALM). These loaded NPs obtained by the solvent evaporation-emulsion technique, significantly enhanced ALM secretion of IL-12 (2.9-fold), TNF-α (16-fold) and INF-γ (23-fold) compared to controls over 24 h; and doubled reactive oxygen and nitrogen species generation over 6 h. NPs could deliver 4-fold more rifampicin into ALM than free rifampicin solution.

Fluoroquinolone antibiotics, such as moxifloxacin (MX) and gatifloxacin, are also active against *M. tuberculosis* showing lower minimum inhibitory concentration (MIC) values than RIF, ciprofloxacin (CP) or levofloxacin (LV). MX-loaded poly(butyl cyanoacrylate) (PBCA) NPs [15] were obtained by anionic polymerization of *n*-butyl-2-cyanoacrylate in the presence of MX. The antimicrobial efficiency of the resulting NPs was tested against *M. tuberculosis* (H37Rv). Encapsulation of MX within NPs demonstrated improved antibiotic uptake and retention by macrophages resulting in an enhanced activity of the drug.

These two antibiotics were also used in other treatments. MX was loaded into positively charged gelatin NPs obtained by a modified two steps de-solvation technique [16]. In this case, NPs were ocularly delivered and the drug was released in a controlled manner in the corneal eye layer. In general, this administration route is widely applied in nanotechnology to obtain higher bioavailability and prolonged ocular residence time of therapeutics [17]. The behaviour of the gelatin-based nanosuspension on the corneal eye surface of rabbits was assessed against *S. aureus* (NCIM 2079) and *B. subtilis* (NCIM 2063) infections. The authors conclude that this suspension was more effective than the commercially marketed product MoxiGram^®^. MX antibiotic was also encapsulated into Ch–dextran sulfate (DS) NPs [18] and the efficiency of the formulation was tested in vitro and ex vivo against *S. aureus* and *P. aeruginosa*. The results revealed that the use of NPs exhibits a prolonged drug release profile with significantly high transcorneal permeation and high corneal retention. Another example is the gatifloxacin-loaded NPs of 50:50 Eudragit^®^ RL and RS mixture which were prepared via nanoprecipitation or double emulsion techniques. The antimicrobial activities of these NPs were tested against *E. coli*, *P. aeruginosa*, and *S. aureus*, showing prolonged antimicrobial effect and prolonged residence time in the eye [19]. PLGA NPs loaded with sparfloxacin (fluoroquinolone antibiotic) were also utilized for ophthalmic delivery [20]. These NPs were prepared by the nanoprecipitation technique and their activity was tested against *P. aeruginosa* using the cup-plate method. They present improved precorneal residence time and ocular penetration as well as higher antibacterial effectiveness than a commercial formulation (brand name unknown).

Regarding LV, Hadinoto’ group prepared LV-loaded polymeric NPs made of PCL or PLGA polymers by either the nanoprecipitation or the emulsification-solvent evaporation method and tested their antibacterial efficacy against *E. coli* K-12 (W3110, CGSC) biofilm cells [21]. They reported that for a successful therapy against biofilm infections, a biphasic release profile is required; a fast antibiotic release at the beginning that ensures a high initial antibiotic concentration followed by a slower extended release which sustains a sufficiently high antibiotic concentration to inhibit biofilm growth and minimize exacerbation. In this study, the loading capacity of the NPs was 0.30%–1.10% *w*/*w*, while the release was found to be 40% the first day and approximately 75% after six days. Such a biphasic release profile was efficiently able to suppress the biofilm growth for up to four days. This approach gives advantage of using reduced doses of antibiotic with decreased associated toxicity. The same group also developed a method to increase the encapsulation efficiency of highly water soluble drugs during the emulsification-solvent evaporation method. Specifically, lecithin was included as a drying adjuvant in the aqueous phase. This resulted in a two-fold improvement in the encapsulation efficiency and loading of LV drug (i.e., 23% and 2.3% *w*/*w*, respectively) compared to the previous method lacking the drying adjuvant [22]. In this work, the antimicrobial efficiency was tested against *P. aeruginosa* biofilm cells. The NPs were lyophilized, reconstituted and spray-dried. Under these conditions, 99.999% of the biofilm cells were killed (initial concentration 10^8^ CFU/peg) after 6 h of exposure to the NPs, with very few cells surviving in the biofilm during the burst release (3 log (CFU/peg)). However, the surviving cells were able to re-establish the biofilm during the slower release stage, reaching 5 log (CFU/peg) cells within 24 h. As result, 99.9% of the biofilm cells were eradicated after one day. They have also analysed the effect of phosphatidylcholine lipid on the encapsulation of LV in PLGA NPs obtained by the emulsion-diffusion-evaporation method [23]. Compared to polymeric NPs, lipid-coated NPs are, in general, more stable. The presence of the lipid gave slower antibiotic release rates but did not improve their biofilm affinity. These NPs exhibited higher antibacterial efficacy against *P. aeruginosa* biofilm cells, but not against planktonic cells detached from the biofilm matrix. This group has also prepared an antibiotic-NP complex or nanoplex by self-assembly amphiphile-polyelectrolyte complexation process between a cationic drug, ofloxacin (OFX) (fluoroquinolone antibiotic) or LV, and the anionic dextran sulfate [24]. They achieved high drug loading ranging from 60% to 80%. Moreover, MIC values of the OFX and LV-nanoplexes tested against *P. aeruginosa* (PAO1 ATCC) planktonic cells were 2.0 and 0.5 µg/mL, respectively, which are comparable to those of non-encapsulated antibiotics.

Polycaprolactone (PCL) is another biodegradable and biocompatible polymer which can be used for antibiotic encapsulation. This polymer was also used by Hadinoto et al. to encapsulate LV. The antibacterial efficiency of the resulting NPs was then evaluated against *E. coli* biofilm [25]. They were able to eradicate 99.9% of the *E. coli* biofilm cells.

Other authors have also encapsulated LV in PLGA NPs by a modified emulsion-diffusion-evaporation method using sucrose and mannitol as lyoprotectants [26]. Mannitol achieved the best performance in terms of less aggregation, higher stability and superior redispersability of the NPs. After a single oral administration of LV-NPs in mice, drug release into the blood plasma was sustained up to 4 days. In contrast, the release of the free drug was completed within 24 h. No signs of toxicity were found in mice after two weeks of treatment with the encapsulated drug. In another study, LV-encapsulated PLGA NPs using 1% *w*/*v* mannitol were evaluated for their in vitro drug release profile and ex vivo transcorneal permeation [27]. Microbiological efficacy of this system was tested against *S. aureus* using the cup-plate method, revealing that the NPs were retained for longer times and drained out from the eye very slowly compared to the marketed formulation. Besides, these NPs did not show any irritant effects on the application site and have a shelf life of up to 2 years.

PLGA alone or in combinations with Eudragit^®^ RLPO or RS30D, with or without 1,2-dioleoyl-3-trimethylammonium-propane chloride salt (DOTAP) (a positively charged transfection-inducing agent), were used to prepare NPs with increased entrapment efficiency of LV [28]. In addition, either formulations containing Eudragit^®^ RLPO or RS30DD with DOTAP, presented higher antibacterial activity against Gram-negative bacteria *E. coli* and *P. aeruginosa* in comparison to free LV solution.

LV were also loaded into cholesterol-bearing hyaluronic acid nanohydrogels prepared by nanoprecipitation [29]. The antimicrobial activity of these nanohydrogels was tested against Gram-negative *P. aeruginosa* (PAO1), and Gram-positive methicillin susceptible *S. aureus* (MSSA) (ATCC 6538P) and MRSA (USA300-0114). They exhibited higher efficiency compared to free antibiotic. These materials also showed that they can be freeze-dried, stored up to 6 months and reconstituted without any loss of their antimicrobial activity.

**Figure 1:**
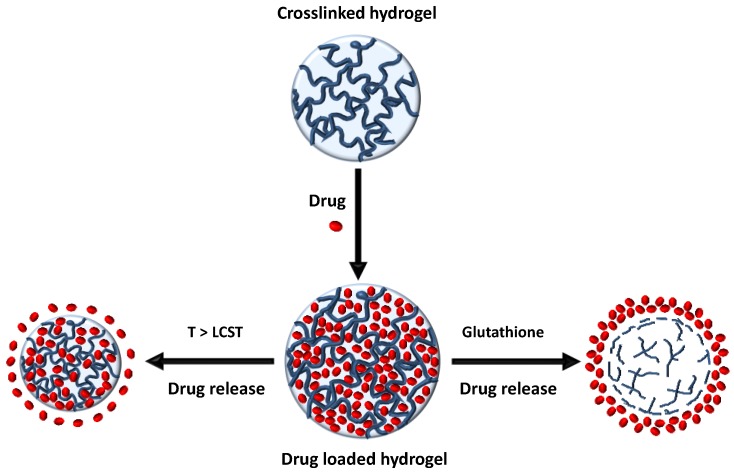
Schematic structure of thermo-responsive and biodegradable hydrogel and the process of drug loading and release from thermo- and reduction-responsive hydrogel after morphological transformation by glutathione.

It is well-known that the use of stimuli-responsive polymeric materials is fundamental for controlled drug delivery systems. This is because they can significantly change their drug release rates in response to a stimulus such as the local pH or temperature in the body. Probably, the most widely investigated stimuli-responsive polymer is poly(*N*-isopropyl acrylamide) (PNIPAM). Jones et al. introduced chlorhexidine diacetate into a hydrogel of NIPAM by immersion in a drug solution at 20 °C (below its LCST: lower critical solution temperature) [30]. In vitro this system was able to eliminate 10^8^ CFU of *Staphylococcus epidermidis* (NCTC 10519) in only 15 min. Recently, thermo-responsive biodegradable hydrogels based on NIPAM and two biodegradable crosslinkers, PCL-dimethacrylate and bisacryloylcystamine, have also been loaded with LV [31]. It was shown that LV release in PBS buffer was less than 30% of the total amount of loaded drug within one month. However, the drug release can be stimulated upon exposure to low concentrations of the reductant glutathione with complete release of LV by 120 h (see Figure 1). In addition, the degradation of these hydrogels, after incubation in glutathione-PBS solution, took place over two months.

Guanidine-containing polypeptide composed of poly(γ-glutamic acid) and arginine (Arg) can self-assemble into colloidal NPs at pH lower than 3.0, and their morphological changes can be reversibly switched by elevating the pH of the colloidal suspension. By this way, the release of amoxicillin from these NPs was reduced at pH 2.5 (gastric fluid, fasted state) and 4.5 (the gastric mucosal surface), but the antibiotic was rapidly released from the NPs at pH 7.0 (the sites for *Helicobacter pylori* infection) [32].

Cyclodextrin (CD)-based polymers have also been used for drug delivery. These cyclic oligosaccharides possess a hydrophilic outer surface (C–OH groups) and a characteristic hydrophilic apolar cavity (C–O–C and C–H bonds) able to form reversible complexes with hydrophobic drugs. For example, LV as well as aspirin and acetaminophen were included into three CDs, namely α-, β- and γ-CD, as part of crosslinked CD-polyurethanes based on toluene-2,4-diisocyanate (TDI) [33]. The results concluded that the β-CD-based polymers have the highest sorption capacities. Hernandez-Montelongo et al. [34] prepared nanoporous silicon particles which were functionalized by in situ polymerization of cyclodextrin with citric acid. Ciprofloxacin (CP), another fluoroquinolone antibiotic, was used as a model drug to analyse its release profile from cyclodextrins. These CD-nanocomposites presented higher drug loads and better controlled release than chemically oxidized nanoporous silicon. Consequently, CD provides enhanced controlled release. In another study, encapsulation of CP in pullulan-PCL core-shell nanospheres was achieved at 35%–40% by weight [35]. Under in vitro test conditions, approximately 20% of CP was released within the first 4 h, with additional slow release over 10 days. In addition, the NPs presented antibacterial activity against *E. coli* and were not toxic against human cell lines.

In other studies, PLGA NPs prepared by nanoprecipitation method were loaded with azithromycin (AZ) [36] or clarithromycin (CL) [37] macrolide antibiotics using different antibiotic concentrations. The loaded NPs were more effective than the free drugs against Gram-positive *S. aureus* (PTCC 1112) and Gram-negative *Salmonela typhi* (PTCC 1609). In fact, the loaded NPs showed identical antibacterial effect than 1/8 the concentration of the pure antibiotics. AZ antibiotic has also been loaded into Eudragit^®^ RS100 nanobeads and nanofibers prepared by the electrospinning technique [38]. Authors employed different drug/polymer ratios and various solution concentrations, and compared the drug release profile in between systems. However, the anti-microbicity of the resulting materials was not assessed in this work. Toti et al. [39] demonstrated that encapsulation of RIF and AZ in PLGA NPs enhances the effectiveness of the antibiotics by reducing microbial burden against *Chlamydia trachomatis* serovar K (UW-31) in HEp2 infected cells (Human lung Epithelial cells). Combination of both antibiotics resulted more effective than the use of the individual drugs.

Molecular imprinting technique and precipitation polymerization have been used to prepare AZ-imprinted poly(methacrylic acid-*co*-ethylene glycol dimethacrylate) NPs [40]. These NPs presented higher AZ loading capacity in comparison with non-imprinted NPs and lower cytotoxicity against L929 fibroblast cells. Previously, molecular imprinted polymers were prepared by polymerization of methacrylic acid and the crosslinker 2-ethyl-2-(hydroxymethyl)propane-l,3-diol in the presence of LV, CP, OFX and sulfamethoxazole (sulfonamide antibiotic) (SF) [41]. The highest bounding capacity was found for LV and analogous, CP and OFX, whereas SF showed the lowest loading.

Chitosan/poly(lactic acid)/tripolyphosphate (Ch/PLA/TPP) NPs have been successfully prepared for encapsulation of AZ. The highest entrapment efficiency obtained was above 85% and these NPs presented sustained and controlled drug release in vitro. Moreover, the Ch/PLA/TPP NPs showed low cytotoxicity when co-cultured with HeLa and HEK293T (Human Embryonic Kidney) cells [42].

β-Lactam antibiotics such as penicillin derivatives, cephalosporins, monobactams, and carbapenems, have been extensively employed in the clinical treatment of many types of bacterial infections. However, their effectiveness is significantly compromised in bacteria that produce β-lactamase enzymes, which hydrolyze the β-lactam ring to an inactive ring-opened product. Turos et al. [43] prepared a variety of penicillin-derivative acrylate monomers to obtain NPs by emulsion polymerization. MIC values of NPs were determined against *S. aureus* (ATCC 25923) and MRSA (ATCC 43300) by broth dilution assays. The results indicated that the penicillin G-conjugated NPs were significantly more active than the 6-aminopenicillanic acid-containing NPs; and that the type of linkage used to covalently attach the drug moiety to the polymer backbone significantly alters the activity of the antibiotic. Moreover, the NPs carrying the antibiotic covalently attached were more effective than those non-bounded, whilst not being cytotoxic to human dermal fibroblast cells. They also synthesized polyacrylate NPs by emulsion polymerization in which a *N*-methylthio β-lactam antibiotic was covalently conjugated onto the polymer [44]. The resulting emulsions displayed potent antibacterial behaviour in vitro against *S. aureus* (ATCC 25923) and MRSA (ATCC 43300) without showing toxicity towards human dermal fibroblasts. They also prepared glycosylated polyacrylate NPs with covalently bound *N-sec*-butylthio β-lactam and penicillin as well as CP [45]. Microbiological tests showed that the NPs bearing *N-sec*-butylthio β-lactam and ciprofloxacin have powerful activities in vitro against *S. aureus* (ATCC 25923), MRSA (ATCC 43300) and *Bacillus anthracis*, while on the other hand the penicillin-bound NPs do not display any antimicrobial activity. Very recently, they have extended this approach to obtain polyacrylate NPs with antimalarial activities against chloroquine-resistant *Plasmodium falciparum* (K1 strain), which causes malaria [45]. As shown, unique properties can be acquired through conjugating antibiotics to polymers such as protecting the antibiotics from degradation or stabilizing sensitive molecules. These polymeric antibiotics allow controlled and sustained drug release whilst enhancing the antimicrobial activity of the drug [46].

Glycosylated NPs based on a poly(*n*-butyl acrylate) (pBA) core and a poly(*N*-2-(β-d-glucosyloxy)ethyl acrylamide) (*p*-(NβGlcEAM)) or poly(*N*-2-(β-d-galactosyloxy)ethyl acrylamide) (p(NβGalEAM)) shell were prepared by nanoprecipitation [47]. Encapsulation and release experiments of ampicillin (penicillin derivative) (AM) revealed that NPs were able to load the antibiotic up to 47%. The release was slow and followed a constant zero-order kinetic, reaching approximately 56% after 21 days. These AM-glycoNPs were effective against *S. aureus* (3R7089 strain Oxford/ATCC9144), *S. epidermidis* (laboratory strain from clinical isolate) and *E. coli* K-12 (W3110/ATCC 27325) bacteria.

In another example, encapsulation of cefamandole nafate (a second generation cephalosporin) into bovine serum albumin (BSA) and polyallylamine (PALA) NPs was performed by the water-in-oil single emulsion technique and posterior crosslinking reaction with glutaraldehyde. These NPs were incorporated into carboxylated-polyurethane (PU) polymer to obtain active films [48]. These NP-drug loaded PUs were found to be active against *S. epidermidis* (ATCC 35984) without being detrimental towards Hp-2 cells growth.

Other kind of potent antibiotics that have also been applied in drug delivery are the aminoglycosides, such as gentamicin (GT) or tobramycin (TOB). PLGA NPs carrying TOB were obtained by a modified emulsion-solvent diffusion technique and subsequently embedded in an inert microcarrier made of lactose. It was shown that the presence of hydrophilic polymers such as poly(vinyl alcohol) (PVA), alginate (Alg) and Ch, helps to optimize the surface and bulk drug release properties of the loaded NPs. In particular, the use of alginate (Alg) allows efficient TOB entrapment into the NPs and its release up to one month along with significant in vitro antimicrobial activity against *P. aeruginosa* bacteria. Besides, in vivo biodistribution studies showed that while the PVA-modified Alg/PLGA NPs were able to reach intro deeper lung tissue, the Ch-modified NPs were found in great amounts in the trachea, bronchus, bronchioles and alveolar ducts, covering the lung epithelial surfaces [49].

**Figure 2:**
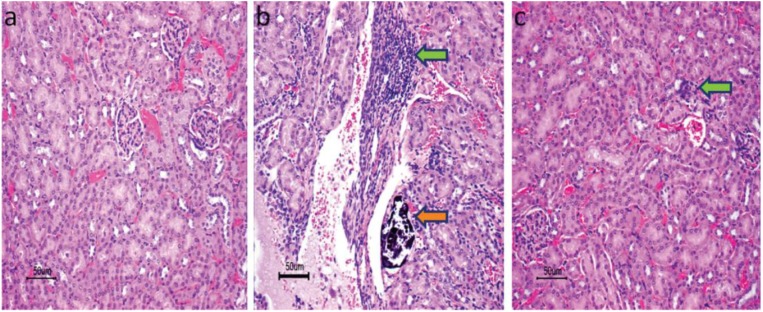
Histopathological microscopic images of kidney tissues of AJ-646 mice euthanized 5 days after intraperitoneal administration: (**a**) untreated control group; (**b**) mice group treated with 15 mg/g body weight with free gentamicin: Minimal to mild lymphocytic inflammation (**green** arrow) associated with mineralized deposits (**orange** arrows); (**c**) mice group treated with 15 mg/g body weight with core-shell nanostructure: typically unremarkable to rare small peri-glomerular aggregates of mononuclear cells (arrow). The tissues were hematoxylin and eosin stained. Scale bar of 50 μm. Reproduced from [50]. Copyright Dove Medical Press Ltd., 2009.

Block copolymers based on poly(sodium acrylate) (PAANa) and Pluronic^®^ F68 (PEO-*b*-PPO-*b*-PEO), PAANa-*b*-(PEO-*b*-PPO-*b*-PEO)-*b*-PAANa (being PEO and PPO poly(ethylene oxide) and poly(propylene oxide), respectively), were used to form nanostructures of PEO-*b*-PPO-*b*-PEO shells and PAA cores able to entrap the antibiotic gentamicin (GT) [50]. The NPs were tested in vitro and in vivo against *Salmonella enterica typhimurium* (wild type) and the findings show a significant reduction of the bacterial population in mice. In addition, the results demonstrate an improvement in the amount and rate of uptake by macrophages there by reducing the toxicity of encapsulated GT in comparison to free GT (see Figure 2).

This antibiotic was also incorporated as part of self-nanoemulsifying formulations in order to obtain drug-loaded polymer NPs. Specifically, the formulations included poly(ethylene glycol) (PEG) 4000 as PEGylated self-assembly system, mixtures of soybean oils and also a combination of Kolliphor^®^ EL and Kolliphor^®^ P188 as surfactants, and Transcutol^®^ HP as co-surfactant [51]. Indeed, these formulations are isotropic mixtures of natural or synthetic oils, surfactants and co-surfactants, which spontaneously emulsify when exposed to gastrointestinal fluid forming oil-in-water nanoemulsions [52]. The effectiveness of these formulations was proven against Gram-positive *S. aureus* and *B. subtilis*, and Gram-negative *E. coli* and *Klebsiella pneumoniae* bacteria by using the agar diffusion method. The inclusion of GT on these formulations decreased the GT nephrotoxicity and ototoxicity, although in vivo tests were not performed in this study.

The non-biodegradable positively charged polymer Eudragit^®^ RL 100 was used to prepare amphotericin B (polyene antimycotics)-loaded NPs by the solvent displacement or nanoprecipitation method [53]. Their antifungal activity was demonstrated against the bacterium *Fusarium solani* by the disk diffusion method and any eye irritation was observed in in vivo tests. Clavanin, an antimicrobial peptide, was also nanostructured using Eudragit^®^ L 100-55 and RS 30D solution [54] achieving an encapsulation efficiency of 98%. These NPs when tested against *S. aureus* (ATCC25923), *E. coli* (ATCC8739), *K. pneumoniae* (ATCC13885) and *P. aeruginosa* (ATCC 27853) bacteria demonstrated significant antibacterial efficiency both in vitro and in vivo.

Liu’s group [55] have extensive analysed enzyme-responsive polymeric vesicles for bacterial strain-selective delivery of antimicrobial agents. Penicillin G amidase (PGa) and β-lactamase (Bla) were selected as target enzymes considering that both enzymes are associated with bacterial resistance. These polymeric vesicles were self-assembled from amphiphilic block copolymers composed of a hydrophilic PEG block and a hydrophobic block bearing enzyme-cleavable self-immolative side linkages. During vesicle formation, antimicrobial agents (i.e., deprotonated quinupristin/dalfopristin (Synercid) hydrophobic bacteriostatic antibiotics; GT; vancomycin (VC); CP; Parasin I, a macromolecular hydrophilic antimicrobial peptide; SAMP1: P(DMAEMA_0.93_-*co*-BMA_0.07_)_22_, a synthetic antimicrobial copolymer where DMAEMA and BMA are 2-(dimethylamino)ethyl methacrylate and butyl methacrylate, respectively) were loaded into either hydrophobic bilayers or aqueous interiors. All the systems were shown to be effective against *P. aeruginosa* (MH340) and MRSA (MRSA252). Besides, these formulations increased the structural stability of the antibiotics in addition to reduce their side effects (diminished hemolysis and toxicity towards HeLa cells, a human cancer cell line). Moreover, Bla-degradable polymeric vesicles were able to enhance the wound healing process in an in vivo murine model.

It has been found that in many of the publications related to antibiotic delivery, the activity of the systems was not tested because the action of these antibiotics is assumed and manifest. Here, we have shown different works where the activity of antibiotics can be modified. In the last five years it has been revealed that the synergy between diverse antibiotics or systems with different mechanisms of action enhances the final activity [11]. This is the case of ampicillin-silver nanocomposites prepared by photoreduction of silver acetate in the presence of poly-(6-[3-(2-(3-aminopropylamino) ethylamino)propylamino])-(6-deoxy)-*b*-CD polymer [56]. The combination of AM and Ag-polymer nanocomposites reduced the MIC values against *E. coli* and the Gram-positive *Kocuria rhizophila* bacteria.

#### 2.1.2. Wound Healing or Dressing

In the design of a successful wound bandage it is necessary to consider the characteristics of the wound type, wound healing time, as well as the physical, mechanical, and chemical properties of the bandage. With a deeper understanding it will be possible to achieve higher healing rates and better aesthetic repair of the wound [57].

Electrospinning is a simple and low-cost method for manufacturing nanoscale polymer fibres [58] and is a very commonly technique in the biomedical field for preparing wound dressings. Chitosan is a non-toxic, biocompatible, biodegradable and hemostatic material with the ability to stimulate wound healing and show inherent antimicrobial properties. For all these reasons, Ch is extensively used to prevent or treat wound and burn infections and not only because of its intrinsic antimicrobial properties, but also by virtue of its ability to deliver extrinsic antimicrobial agents [59,60]. For example, chitosan–pectin–TiO_2_ dressing was prepared by blending Ch with titanium dioxide or titania (TiO_2_) NPs in solution and being electrospun into fibres. While the titania NPs endow antibacterial effectiveness against Gram-positive and Gram-negative bacteria, cell growth and high corrosion resistance; pectin, a natural prophylactic substance, provides protection against poisoning by toxic cations along with styptic and curing effects [61]. In another work, Ch was successfully electrospun with nanocellulose, a biocompatible, biodegradable and sustainable biomaterial which has found extensive application in biotechnological areas such as tissue engineering, drug delivery and wound dressing [62]. These membranes were successfully able to kill 99.9% of the bacterial population when tested against *S. aureus* and *E. coli*. The results also revealed that wounds healed more rapidly when treated with Ch-nanocellulose dressings than when treated with only fibrous nanocellulose or the commercial Tegaderm™ hydrocolloid or transparent film wound dressings. As a matter of fact, nanocellulose was also used to attach CD that incorporates carvacrol (2-methyl-5-(1-methylethyl) phenol), a plant essential oil with good antimicrobial properties [63] demonstrating its versatility as wound dressing material. The activity of the surface was tested against *B. subtilis* using a standard dynamic shake flask method. Due to the carvacrol inclusion into the CD, the active compound presented a slow release and its activity was maintained for up to 48 h. Nanocellulose wound dressings were developed where they were impregnated with silver NPs due to the latter’s potent antimicrobial activity. The efficacy of this construct was demonstrated against *S. aureus* and *E. coli* bacteria by the diffusion test and the colony counting method [64].

Dutta’s group has developed various wound dressing materials also based on chitosan. They prepared blends of Ch, TiO_2_ and poly(*N*-vinylpyrrolidone) (PVP) [65], a synthetic polymer with good biocompatibility. The resulting membranes showed great antimicrobial activity against *S. aureus*, *B. subtilis*, *E. coli*, and *P. aeruginosa* bacteria without compromising the cell viability of L929 mouse fibroblast cells and NIH3T3 mouse embryonic cells, as confirmed by the Alamar blue assay. Moreover, the wound closure rate was effective in the nanocomposite-treated wounds in full-thickness wound model of adult male albino rats compared to control experiments. This group also prepared blends of Ch, PVP and silver oxide, which presented similar characteristics to the previous system but with the added advantage of film transparency making possible to monitor the wound [66]. The silver NPs were synthesized using carboxymethyl chitosan as both reducing agent for silver ions and as protecting agent for the synthetized NPs. Posterior electrospinning of the mixture with PEO resulted in nanofiber mats which demonstrated excellent antimicrobial activities against *S. aureus* (ATCC 25923), *P. aeruginosa* (ATCC 27853) and *E. coli* (ATCC 25922) bacteria and *Candida albicans* (ATCC 10231) fungi. Moreover, the silver-containing nanofiber mats were more active in comparison to nanofibers without silver NPs and silver NPs alone [67]. Furthermore, quaternized Ch was electrospun with PVP and then photocrosslinked to become water-stable. These mats achieved a 5 log reduction in CFUs in 60 and 120 min against *S. aureus* 749 and *E. coli* 3588 bacteria, respectively [68]. Combination of two antimicrobial systems is another approach used for obtaining antimicrobial fibres. Inorganic NPs such as TiO_2_, silver (Ag) or zinc oxide (ZnO) NPs were incorporated into gellan gum and the nanocomposites were cast into films. A mixture of chitosan with LV was placed as a double layer on top of these films [69]. By this methodology, the materials exhibited antibacterial activity against *E. coli* (JM 109), while allowing the growth of L929 cells. Chitosan was also electrospun with PVA [70,71], PLGA [70,72] and silk fibroin [73] to improve its processability.

Other polysaccharides and proteins have also been used to fabricate wound dressings [74,75]. Fibres of gelatin with silver NPs were prepared by electrospinning [76]. The release of these NPs in acetate buffer (pH 5.5) occurred rapidly in the first hour and then steadily increased; while in simulated body fluid (pH 7.4) there was a gradual increase with time. The antimicrobial effectiveness was demonstrated against *E. coli* (ATCC 25922), *P. aeruginosa* (ATCC 27853), *S. aureus* (ATCC 25023), and MRSA (ATCC 20627) bacteria. In another study, PVA was blended with cellulose nanowhiskers to obtain hydrogels for wound dressing by the freezing-thawing technique [77]. In this case, no antimicrobial system was incorporated so these systems did not actively kill microbes. However, due to their improved mechanical properties and water vapour transmission rate, these hydrogels can be considered as a good barrier against different microorganisms. On the other hand, the preparation of PVA nanofibers containing Ag NPs by electrospinning, greatly improved the antimicrobial effect of the fibres. A CFU reduction of >99.9% was achieved after incubation with *S. aureus* or *K. pneumoniae* bacteria for 18 h [78].

Jalvandi et al. [79] loaded LV into mesoporous silica NPs (MSN), which were subsequently incorporated in the core region of PCL nanofibers via core–shell electrospinning. The antibiotic release from these fibres was more gradual than when released from PCL-LV and PCL-LV core-shell nanofibrous mats. Moreover, the antimicrobial activity against *E. coli* (ATCC 4352) bacteria was more effective over time, with a reduction in bacterial population occurring over a span of 14 days instead of only 7 days as was the case in the controls. The results showed that the physical adsorption of a drug on a nanostructure and distribution of this nanostructure along the nanofibers provides more effective sustained drug release. PCL was also electrospun with PVP containing extract of *Tecomella undulata*, a medicinal plant widely known for its traditional medical applications including wound healing capability. Extract-loaded PCL/PVP mats were able to efficiently inhibit the growth of *S. aureus* (ATCC 933), *P. aeruginosa* (MTCC 2297), and *E. coli* (IP-406006) bacteria [80]. Other synthetic polymers such as polyurethane, polyacrylonitrile, poly(acrylamide)/poly(vinyl sulfonic acid sodium salt) or poly(sulfobetaine methacrylate) have also been used for developing wound dressings [81,82,83,84]. For example, fluoroquinolones, CP and norfloxacin (NR), were attached to polyphosphazenes by chemical modification using amino acid esters (alanine, glycine, phenylalanine) as chain extenders [85]. These components were then electrospun into nanofibers. Polymers that contained alanine ethyl ester or glycine ethyl ester co-substituents showed faster degradation rates and antibiotic release profiles than the polymers that contained phenylalanine ethyl ester co-substituents. Also, CP and NP released in the hydrolysis media showed a MIC of ≤0.45 and 0.58 µg/mL, respectively, as determined by consecutive double dilutions.

All these examples mentioned above highlight the benefits of using various polymeric materials for wound dressing applications. In particular, those able to regulate the antibiotic release are strong promising candidates for these applications.

#### 2.1.3. Sutures and Prosthesis

Microbial cells may attach and proliferate on any artificial surface when they enter in contact with them, overall if these are located within a moist environment. Under these conditions, the bacteria population rapidly increases on the surface and eventually builds up a biofilm, which consists in a complex organization of polysaccharide matrix and embedded bacteria among many other components. This biofilm allows microbial cells to survive under harsh conditions and to be up to 1000 times less susceptible to most antibiotics and other biocides. One of the main approaches to prevent the biofilm growth and consequently, to inhibit the spread of microbial infections, is the use of antimicrobial surfaces [3,5,86,87]. Such surfaces either repel microbes, so that they cannot attach to the surface, or kill the microbes in the surrounding area.

Surgical sutures are probably the most consumed medical devices for wound closure. During the surgery, sutures as well as implants can be exposed to microorganisms present in the environment leading to contamination, bacterial biofilm formation and, therefore, infections [88]. Infections cause a great part of the postoperative morbidity and mortality in hospitals. As mentioned, antimicrobial polymers are a potential alternative since they can be shaped into wound sutures, artificial tendons, bone cements or medical packaging among others.

Polyamides are by far the mostly applied material in sutures. However, antimicrobial commercial products are only a few, i.e., PLGA, polydioxanone (PDO) and poly(glycolide-*co*-ε-caprolactone) (PGCL) with triclosan, PDO, PGCL and poly(glycolic acid) (PGA) with chlorhexidine diacetate [88]. It was demonstrated that antimicrobial sutures lead to a significant decrease of the incidence of surgical site infections [89]. Having this in mind, researchers have been focused on two strategies; the modification of the surface via chemical or physical methods. In addition, these surface modifications occur only at the nano/microscale so that the bulk properties of the suture are maintained.

Physical modification typically entails polymer impregnation or blending with different antibiotics [90,91,92] and/or antiseptic substances, i.e., triclosan, chlorhexidine (CHX), silver and so on [93,94,95]. Although drug-loaded coatings prepared in this way can provide good localized drug concentration, the variable loading efficiency and release kinetics limit their use. Drug incorporation within suture matrices is an alternative strategy. However, suture strength can be compromised and it is required of severe fabrication conditions for suture-strength enhancement. More mechanically robust sutures were very recently described by Padmakumar et al. [96] who prepared electrospun core–shell yarns with a central PLA core and a drug-eluting PLGA shell. Coatings or blends can also be made with polymers with intrinsic antimicrobial properties. This is the case of PLGA coated with an amphiphilic polymer, poly[(aminoethyl methacrylate)-*co*-(butyl methacrylate)], which was bactericidal against MRSA (ATCC 33591) bacteria. The sutures containing the antimicrobial polymer killed *S. aureus* (ATCC 25923) bacteria more efficiently (3 log-reduction at 2.4 wt %) than PLGA sutures (Vicryl^®^ Plus) which contains triclosan as antimicrobial agent (0.5 log-reduction) [97]. More recently, a *N*-halamine-modified PGA multifilament obtained via layer-by-layer technique has been described [98]. Since *N*-halamines have a high efficacy in killing bacteria, the resulting sutures were able to inactivate *S. aureus* (ATCC 6538) and *E. coli* O157:H7 (ATCC 43895) bacteria within 30 min of contact time.

PGA sutures with long-term antimicrobial activity were obtained by dip-coating into two different polymeric silver nanocomposites. These two solutions were obtained by dissolving silver nitrate in modified hyperbranched polylysine solutions in toluene, followed by reduction with L-ascorbic acid and generation of highly stabilized silver NPs. These coated sutures showed high activity against *S. aureus* cells for more than 30 days and non-cytotoxicity against L929 mouse fibroblast cells [99].

As mentioned above, the chemical modifications performed on the material surface may also greatly modulate its antimicrobial properties. For example, Ivanova’s group demonstrated that black silicon that contains high aspect ratio nanoprotrusions on its surface, also possesses remarkable surface properties such as high hydrophobicity and strong biological activity at cellular level [100]. And also the nanostructuration induced by oxygen plasma treatment onto non-absorbable monofilaments of polypropylene (PP) (Premilene^®^) and poly(ethylene terephthalate) (PET) (Miralene^®^) and absorbable monofilament sutures of PDO (Monoplus^®^) and modified PGA (Monosyn^®^), demonstrated to be effective against *E. coli* K12 wild type (DSM A498, ATCC 23716) bacteria [101].

**Figure 3:**
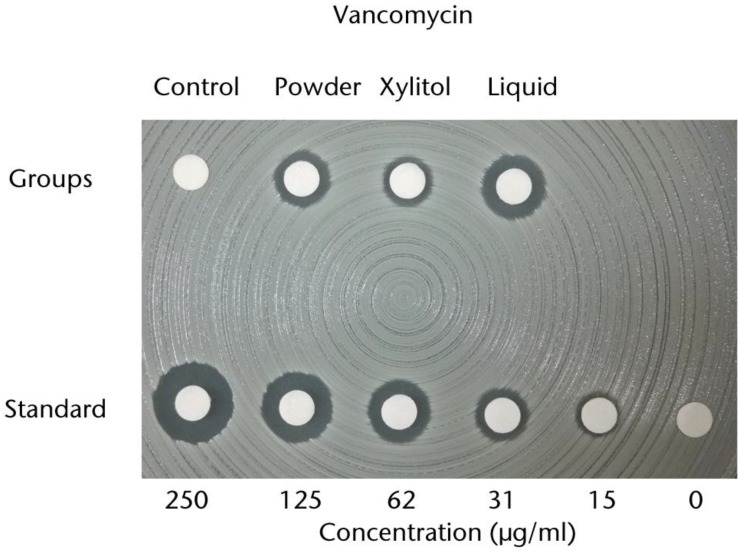
Charts showing antibacterial activity of samples eluted from bone cement with different preparations (antibiotic preparations added to bone cement: powder vancomycin, the inert filler xylitol/powder vancomycin, liquid vancomycin), as determined by the agar-disk diffusion bioassay. Bone cement without antibiotics served as control. The data are presented in terms of growing inhibition of methicillin-resistant *S. aureus* bacteria. The growth was visually compared with standard samples containing different concentrations of vancomycin. Reproduced from reference [105]. Copyright the British Editorial Society of Bone & Joint Surgery, 2014.

These approaches applied in sutures can also be applied in orthopedic and trauma surgery. For example the use of antimicrobial systems for prophylaxis in patients undertaking joint replacement surgery or fracture fixation [102]. Polymeric bone cements, mainly those composed of poly(methyl methacrylate) (PMMA) loaded with gentamicin, have been widely used. However, extensive antibacterial resistance appeared. For this reason, PMMA cement was loaded with other active molecules, such as TB and VC antibiotics (see Figure 3) [103,104,105], with inorganic or metallic NPs as silver, [106,107,108] or with antimicrobial polymeric NPs such as quaternary ammonium polymers [109,110,111].

Nevertheless, other approaches have been utilized in bone implant and bone regenerative medicine. Specifically, the combination of NPs of hydroxyapatite (HA), a calcium phosphate (CaP) compound similar in morphology and composition to the human hard tissues, with polymers possessing osteoconductive properties enhanced the bone formation [112]. In addition, scaffolds of Ch alone or Ch with metallic NPs have been widely applied [93,113,114]. Bioactive nanocomposite scaffolds were also successfully developed using PCL, crosslinked gelatin and HA NPs through layer solvent casting combined with freeze-drying and lamination techniques [115]. In another study, PLGA NPs loaded with nafcillin (β-lactam antibiotic) or LV were obtained by emulsion-solvent evaporation technique, followed by calcium phosphate (CaP) coating via surface adsorption-nucleation method. These NPs were developed to treat osteomyelitis, which is a bone infection mainly caused by various strains of bacteria, being *S. aureus* responsible for 80% of all human cases. If the infection persists, open surgical debridement in addition to antibiotic therapy is normally required. In vitro drug release profile of these synthesized NPs shows a typical biphasic release, which is governed by diffusion and degradation mechanisms, and exhibits a sustained release profile up to 4–6 weeks. The particles inhibit biofilm formation for up to 4 weeks, and disintegrate and deteriorate biofilms completely after 7 days [116].

Many of the examples shown here can be applied in the next section, since the fundamental characteristics are similar [117].

#### 2.1.4. Dental Applications

Dental problems, which range from cavities to gum disease or oral cancer, cause enormous health care costs to the population only considering dental restorations, which is the most common infection. Resin-based dental composites have been widely used in dentistry to restore decayed teeth. These composites have sufficient flexural strength and outstanding aesthetics; but nowadays they also require of antimicrobial characteristics [118].

Monomers containing quaternary ammonium nitrogens were widely applied as part of dental resins as well as many other active ones [119,120,121]. Tiller’s group has used antimicrobial polymers made of poly(2-alkyloxazoline)s for dental materials. They employed a macromeric biocide based on poly(2-methyl oxazoline) with quaternary ammonium end group, which has the ability to render a contact-active antimicrobial material. This polymer, used as additive in a commercial dental adhesive, was able to kill *Streptococcus mutans* cells in the tubuli of tooth and to inhibit bacterial and human collagenases and gelatinases, two enzymes related to tissue destruction in periodontal disease [122]. Beyth et al. also improved dental resins by incorporating polyethyleneimine (PEI) NPs, which enhanced its effectiveness against *S. mutans* bacteria [123]. Other materials such as silica were also modified with quaternary ammonium to fight against *S. mutans* bacteria [124,125].

Silver NPs and mesoporous silica NPs, which are used to incorporate bioactive compounds such as CHX, are also found in dental materials [126,127,128,129]. In fact, CHX was also incorporated into amorphous calcium phosphate (ACP), Ca_3_(PO_4_)_2_ and calcium fluoride (CaF_2_) via a spray-drying technique resulting in dental resin nanocomposites active against *S. mutans* (ATCC 700610) biofilm formation. They reduced the acid production and the metabolic activity of the biofilm by 10–20 times compared to a commercial composite [130]. Moreover, the same group also tested the incorporation of quaternary ammonium dimethacrylate and Ag NPs into a calcium phosphate nanocomposite [131]. The combination of both antimicrobial agents into the same system produced higher activity against *S. mutans* bacteria biofilms than that of either agent used separately. In another studies done by Xu and coworkers [132,133], it was recently found that the addition of 5% of dimethylaminohexadecyl methacrylate in the formulation provokes a 3 log reduction against a wide variety of bacteria, including Gram-positive *S. mutans*, *Enterococcus faecalis* (ATCC 35667), *Enterococcus faecium* (ATCC 4083) and *Parvimonas micra* (ATCC 33270), and Gram-negative *Porphyromonas gingivalis* (ATCC 33277), *Prevotella intermedia* (ATCC 25611), *Prevotella nigrescens* (ATCC 25261), *Aggregatibacter actinomycetemcomitans* (ATCC 43717) and *Fusobacterium nucleatum* (ATCC 25586) bacteria.

Zinc oxide NPs were also blended into dental composites for antimicrobial purposes. In particular the use of 10% in weight of ZnO displayed significant antimicrobial activity and reduced growth of bacterial biofilms against Gram-positive *Streptococcus sobrinis* (ATCC 27352) bacteria [134]. Moreover, lower amounts of ZnO or Ag NPs (1–5 wt %) exhibited potent activity against Gram-positive *S. mutans* (PTCC 1683) and *Lactobacillus* (PTCC 1643) bacteria [134,135,136]. Other antimicrobial agents such as nitric oxide (NO) [137] have been investigated in dental applications. For instance, the ability of NO-releasing silica NPs to kill biofilm-based microbial cells was extensively reported by Schoenfisch et al. [138,139]. In another work, a novel strategy to control plaque-biofilms was freshly reported [140]. In this case, catalytic NPs with peroxidase-like activity were employed to trigger extracellular matrix degradation and to cause bacterial death within acidic niches of caries-causing biofilm. More precisely, the catalytic NPs containing biocompatible Fe_3_O_4_ were able to catalyse H_2_O_2_ decomposition to generate free-radicals in situ that simultaneously degrade the biofilm matrix and rapidly kill bacterial cells. In this way, a >5-log reduction of cell viability of *S. mutans* (ATCC 700610/UA159) bacteria was achieved. In addition, these NPs reduced apatite demineralization in acidic conditions.

### 2.2. Food

Food technology is one of the industrial sectors where antimicrobial polymers play an important role. Nowadays, there is a growing concern regarding not only food preservation and quality maintenance, but also and more important to guarantee food safety for consumers. Food-borne diseases are typically caused by consuming foods or beverages contaminated by pathogens, toxins or chemicals. However, most of these illnesses are infections provoked by pathogens such as bacteria, viruses, fungi and parasites. Some of the most common pathogens include norovirus, *Salmonella*, *Clostridium perfringens*, *Listeria*, *S. aureus* and *Campylobacter*, which can also cause eventual deaths. Currently, much effort has been expended on investigating how to prevent food-borne illnesses, especially in the protection of food from microbial growth during storage. To this regard, antimicrobial polymeric materials are attracting a great interest in this field as presently, most of the food packaging is based on polymeric materials [141].

Over the last decades, novel polymer materials for food packaging, especially based on nanotechnology, have been developed with innovative and more efficient antimicrobial properties. In addition, antimicrobial polymers can be used as food additives and as edible films. In these two latter applications, the polymer should be human-safe suitable for oral administration. Typically, biopolymers such as chitosan and nisin are employed in the food industry. Nevertheless, the antimicrobial products intended for food industry require exhaustive testing. Especially products of nanotechnology need especial care due to the concerns related to the undetermined toxicity of NPs that could be undesired released [142,143]. Next, it will be highlighted the most innovative systems published over the last decade concerning antimicrobial polymeric nanomaterials used in food industry to improve food safety and shelf life.

#### 2.2.1. Food-Packaging

The use of polymeric materials for food packaging has considerably augmented due to their enhanced properties compared to other traditional materials including strength, flexibility and barrier to oxygen, water vapour, carbon dioxide and other food-related compounds such as flavours and taints. Recent advances in the area, especially in the polymer nanotechnology, are improving all these properties even more to address the increasingly more severe requirements in safety and quality of food products during storage and transportation, and to prolong their shelf life [141]. In fact, the use of nanocomposites in food packaging has become one of the most proliferating area of research in the food industry. NPs proportionally possess larger surface area than their corresponding microscale particles, thus favouring the particle-polymer interactions and enhancing their mechanical and processing properties. Besides, the inclusion of NPs adds new functionalities to the material as the capability to release or absorb substances into or from the food or the surrounding; this is known as active packaging. At this moment, most of the active packaging materials are designed for antimicrobial packaging [144,145,146]. These nanocomposite systems are particularly effective against microorganisms because of their high surface area and enhanced reactivity. A wide range of antimicrobial agents have been used or tested in the preparation of antimicrobial nanocomposites for food packaging, including metal NPs (silver, copper), metal oxide NPs (TiO_2_, ZnO, MgO), organically modified nanoclays, natural biopolymers (chitosan) and particles loaded with natural agents (nisin, essential oils) or synthetic compounds (antibiotics, quaternary ammonium salts, organic acids) [147,148]. Each of these antimicrobial compounds exhibits a determined mode of action, such as altering metabolic processes or disrupting the cell membrane. In addition, combinations of more than one antimicrobials incorporated into packaging have also been investigated as very efficient systems. In relation to the polymer matrix, mainly petroleum-based polymeric materials have been used to develop antimicrobial food packaging films including polyethylene (PE), polypropylene (PP), polystyrene (PS), ethylene/vinyl acetate copolymer (EVA), ethylene-vinyl alcohol copolymer (EVOH) and polyethylene terephthalate (PET) [149]. However, recent concerns regarding the environmental impact of plastics have conducted to an increased demand for biodegradable materials such as PLA, PGA, PCL and PVA among others. Particularly interesting are those biodegradable polymers obtained from renewable sources, biopolymers, which include starch, cellulose, chitosan, alginate, carrageenan, soy protein, corn zein, wheat gluten, gelatin, collagen, etc. [150]. Besides, it is of great importance in the food industry the preparation of nanocomposites based on biodegradable biopolymers such as chitosan that possesses antimicrobial activity by itself against many fungi, bacteria, and viruses [151].

In general, antimicrobial food packaging films based on either synthetic or natural polymers can contain antimicrobial agents able to migrate into the food or in contrast, inhibit microbial growth on the food surface by direct contact. This is dependent on the type of antimicrobial NPs incorporated into the matrix and on the nanocomposite preparation method. The antimicrobial activity of these nanocomposites is also greatly influenced by other parameters such as particle size, dispersability and the interaction between the NP and the polymer. Next, the most important nanocomposites systems for antimicrobial food packaging will be discussed.

By far, silver NPs (Ag NPs) are the most studied metal NPs with antimicrobial activity used in nanocomposites films and they are already found in commercially available antimicrobial materials [152]. This is mainly due to their unique physico-chemical properties and particularly strong antimicrobial activity against a broad spectrum of bacteria, viruses and fungi. Although the exact mechanism of action is not clearly known, different modes of action have been proposed for the antimicrobial function of silver NPs that include the release of silver ions and formation of radicals. These species can damage cell membrane and interact with proteins, enzymes or DNA [153]. Metallic silver NPs have been readily incorporated into common non-degradable thermoplastic packaging materials such as PE, EVOH and polystyrene (PS). A summary of selected papers is shown in Table 1.

For instance, low-density polyethylene (LDPE) polymer matrix containing Ag NPs was prepared as packaging material for dried barberry to preserve apparent colour, sensory factors and reduce microbial growth [154]. Ag-LDPE films with 2 wt % of Ag NPs demonstrated antimicrobial activity against meld and total bacteria count with 2.3 and 2.84 log reductions of CFU, respectively. In addition, the incorporation of Ag NPs preserves the red colour of the barberry, brightness, taste and aroma for longer time in comparison with unloaded packaging. On the other hand, nanocomposites containing Ag NPs and made of biopolymers such as chitosan and starch were also prepared. Yoksan et al. developed silver NP-loaded chitosan-starch films for potential application in food packaging [157]. The incorporation of Ag NPs improved properties such as the tensile strength and oxygen gas barrier and provided bactericidal performance against *E. coli* (ATCC 35218), Gram-positive *S. aureus* (ATCC 6538) and *Bacillus cereus* (ATCC 11778), as shown by agar disk diffusion method.

In addition, other types of nanomaterials containing silver ions are commonly used as antimicrobial agents in food packaging films, such as zeolites [158] and nanoclay [159]. Regarding metal oxide NPs, photocatalytic TiO_2_ and ZnO NPs have been extensively exploited for the preparation of antimicrobial packaging films due to their strong antimicrobial activity (Table 1). Basically, their mechanism of action consists in the generation of reactive oxygen species (ROS) when these NPs are irradiated with ultraviolet light. Cerrada et al. [161,162,163,164,165,166] prepared different nanocomposites with antimicrobial activity upon light excitation by incorporating TiO_2_ NPs into food-packaging polymers such as EVOH, PP and PCL. These nanocomposites displayed an excellent performance in killing microorganisms reaching log-reduction of near ca. 8 units upon short irradiation times. Nanocomposites based on ZnO NPs have also demonstrated optimal antimicrobial performance for food packaging applications. For example, the antimicrobial capability of ZnO NPs in LDPE nanocomposites packaging was evaluated in real orange juice samples stored at 4 °C [167]. The nanocomposites containing ZnO NPs were able to extend shelf life (6 log reduction of CFU/mL) of fresh orange juice up to 28 days while maintaining its sensorial parameters.

Furthermore, nanoclays are typically added into polymer matrices to enhance their mechanical and barrier properties, but also they can be modified in order to provide antimicrobial activity. Hong et al. published the antibacterial activity of organically modified montmorillonites containing quaternary ammonium groups [170] and the antimicrobial effect when they are incorporated in polymer films such as PLA and whey protein [171,172]. They demonstrated a bacteriostatic function against Gram-positive *Listeria monocytogenes* (ATCC 19111) bacteria. Besides of the effects of some modified nanoclays by themselves, several works have described nanoclays able to retain antimicrobial agents and control their diffusion from the polymer matrices. For instance, the antimicrobial agent carvacrol was incorporated into wheat gluten/montmorillonite nanocomposites films [173]. It was demonstrated that in the presence of a sufficiently high content of montmorillonite, the system is able to retain the active agents, thus protecting them during the processing stage.

Nanoencapsulation of antimicrobial agents is also an interesting alternative to prepare antimicrobial nanocomposites [174]. Nisin is a cationic peptide produced by *Lactococcus lactis* subsp. with a broad spectrum antibacterial activity against food-borne pathogens. However, nisin is able to interact with various food components and is easily degraded. Thus, its nanoencapsulation can significantly enhance its antimicrobial effectiveness. As an example, nisin was nanoencapsulated in nanoliposomes [175] and then incorporated in hydroxypropyl methylcellulose films. Then biodegradable nano-active films were obtained and can be potentially used as preventive food packaging systems.

#### 2.2.2. Edible Films

Currently, food packaging is based mainly on petroleum-based materials, including PP and PE, which are not sustainable and create tons of plastic waste every year. As previously commented, there is an increasing society’s interest for the use of more natural and biodegradable products in food packaging to reduce waste percentage. Besides, consumers demand safer food with more natural preservative components rather than synthetic additives. This is forcing food industry to develop new packaging strategies that match consumers’ requirements by replacing synthetic packaging and additives by natural components. Edible packaging using edible and biodegradable biopolymers has stated as a promising alternative to synthetic films although this does not mean a total replacement of them [176,177,178,179,180,181,182]. Research on the use of edible films as packaging materials aims at improving food quality and safety with longer shelf life. Such biopolymers principally include polysaccharides (alginate, starch, pectin cellulose and chitosan) and proteins (whey protein, wheat gluten, collagen, zein, soy and casein), which can act as a barrier to control the diffusion of water and gases and can serve as carriers of different food additives such as flavouring agents, antioxidants, vitamins, and antimicrobials. In particular, the use of edible films containing antimicrobial agents has demonstrated to efficiently decrease pathogen growth and prolong the shelf life of a wide range of foodstuff. The most common antimicrobial agents used in edible films are natural compounds including organic acids, essential oils and antimicrobial biopolymers such as chitosan and nisin. Indeed, such as biopolymers with antimicrobial activity, they can be directly used as edible films on food [151].

Several aspects must be considered for the selection of the biopolymers and the antimicrobial agents in addition to their effectiveness against microorganisms. Film-forming behaviour, barrier and mechanical properties and their interactions with other components present on food are also important features. Besides, the incorporation of the active agents into the biopolymers has to be uniform forming homogeneous films, and efficient, by avoiding a rapid diffusion of the substance that results in partial inactivation of the surface. In general, incorporation of antimicrobial NPs in the design of edible films, results in films with improved properties not only related to the antimicrobial activity but also exhibiting better physico-chemical and mechanical characteristics [177,183].

As shown in the previous sections, Ag NPs have been widely used for the preparation of antimicrobial nanocomposites due to their broad-spectrum antimicrobial properties. The incorporation of Ag NPs in the formulation of edible films has also been evaluated. For instance, binary blends of agar and banana were reinforced with Ag NPs in order to prepare edible films with antimicrobial activity [183]. In this publication, Ag NPs were synthesized in the mixture only by the action of banana powder without the need of any additional hazardous chemical reagent. Banana powder in addition to improve water barrier and to have good film forming properties, contains some phytochemicals able to reduce metal ions and produce NPs. The antimicrobial activity of the resulting nanocomposite films was tested against food-borne pathogenic bacteria *E. coli* (ATCC 43895) and *L. monocytogenes* (ATCC 15313). It was demonstrated that the incorporation of Ag NPs provides antimicrobial activity in comparison with the binary blend alone. Besides, composite films showed only bacteriostatic activity against *L. monocytogenes* while exhibited excellent bactericidal activity against *E. coli*.

In another study, Ag NPs were added into pullulan edible films, which is an extracellular polysaccharide produced by the fungal organism *Aureobasidium pullulans* [184]. Besides, ZnO NPs and essential oils from rosemary (*Rosmarinus officinalis*) and oregano (*Origanum minutiflorum*) were also employed as antimicrobial agents. The activity of the resulting composites was investigated in vitro and in situ on meat and poultry products, against the food pathogens *S. aureus* (ATCC 11988), *L. monocytogenes* (ATCC 94229), *E. coli* (ATCC 43895) and *S. Typhimurium* (ATCC 14028). Plate overlay assays demonstrated that these antimicrobial agents added into pullulan films effectively inhibit the pathogenic microorganisms when applied to vacuum packaged meat and poultry products stored at 4 °C for up to 3 weeks. These essential oils and many others obtained from plants have been extensively exploited as natural antimicrobials in edible films to replace synthetic preservatives and achieve safer products for consumers. However, although essential oils exhibit good antimicrobial activity against food-borne microorganisms, their low water solubility limits their applicability in food industry. In fact, edible films are typically prepared from water-soluble polymeric solutions, thus the incorporation of nonpolar essential oils becomes rather difficult leading to non-homogeneous films.

To improve water dispersion of essential oils in the polymeric solution, the preparation of nanoemulsion has emerged nowadays as an interesting alternative. For instance, nanoemulsions of clove bud (*Syzygium aromaticum*) and oregano (*Origanum vulgare*) with diameters of 180–250 nm were prepared by ultrasonication and then dispersed in methylcellulose to obtain antimicrobial edible films [180]. It was shown that the incorporation of essential oils improves film formation in addition to provide them of antimicrobial activity. The use of the nanoemulsions significantly reduced the counts of *Penicillium* sp. (ATCC 2147) and *Aspergillus niger* (ATCC 16404) in sliced bread during 15 days in comparison with those prepared from bigger droplet sizes (diameters of 1.3–1.9 μm). This improved antimicrobial activity achieved when using nanosized droplets is attributed to an increased bioavailability of the encapsulated essential oils that present a higher surface area. In a similar way, alginate based edible films were prepared from nanoemulsions of essential oils containing thyme, lemongrass and sage oil with sizes between 35 and 80 nm [181]. The antimicrobial activity of the edible films containing the essential oils was then studied against *E. coli* bacteria. However, only the thyme-containing film showed a strong antimicrobial effect, reaching 4.71 log reduction after 12 h. In the other samples no evidence of growth inhibition was found. This can be explained by the high activity that exhibit thymol molecules present in the essential oil of thyme [185].

Thymol has also been employed as antimicrobial additive in Ch edible films [186]. Chitosan and its derivatives have been widely investigated as edible coatings due to their biocompatibility, low toxicity, biodegradability and antimicrobial character [151]. However, the antimicrobial activity of Ch depends on various parameters such as its degree of deacetylation and its molecular weight. Besides, Ch activity often decreases when blended with other components such as proteins or lipids, typically used to enhance the mechanical properties or the water vapour permeability of the films. Therefore, antimicrobial compounds such as thymol have been incorporated into the chitosan films to overcome these limitations. Concretely, Ch-tripolyphosphate NPs loaded with thymol were incorporated by inkjet printing in films made of a blend of Ch and quinoa-protein [186]. It was shown that the films containing the NPs exhibited good water vapour barrier and mechanical properties. In comparison to control films, they also showed higher antimicrobial activity against Gram-positive *Listeria innocua* (ATCC 33090) and *S. aureus* (ATCC 25923) and Gram-negative *S. typhimurium* (ATCC 14028), *Enterobacter aerogenes* (ATCC 13048), *P. aeruginosa* (ATCC 27853), and *E. coli* (ATCC 25922) bacteria. This strategy of incorporating antimicrobial agents into edible films by nanoencapsulation has also been employed in many other investigations. In another example, natamycin, which is a natural antimycotic agent approved by the Food and Drug Administration (FDA) of United States and by the European Union (E235), was loaded in PNIPAM nanohydrogels [187]. These nanohydrogels were subsequently added to κ-carrageenan and locust bean gum edible films without almost modifying their main packaging properties. A distinctive feature of this system is that the nanohydrogel can protect natamycin from the surroundings and allow its smart release depending on the environmental temperature.

#### 2.2.3. Food Additives

In addition to incorporating antimicrobial agents in polymeric materials used for food packaging, those antimicrobial compounds can be directly used as additives in food and beverages. The direct addition of antimicrobial compounds has been principally explored in beverages such as juices and milk. Inclusion of these antimicrobial agents in food improves the safety of the products inhibiting the growth of microorganisms and thus preventing from food-borne diseases. Likewise, in edible films, natural antimicrobials have attracted considerable attention in latest years due to the increasing consumer demand on food safety. However, both synthetic and natural agents could cause undesired changes when embedded in the food system, such as loss of the activity of nutrients or modification of the texture. Hence, low concentrations are typically used to not alter the quality of the food products but high enough to inhibit microbial growth. In this sense, nanoencapsulation of antimicrobial agents represents an efficient approach to increase their activity by protecting them from interactions with food ingredients, augmenting its stability and dispersability, and even by selectively locating them in the food areas where the microorganisms preferentially proliferate, for instance in water-rich phases. Nisin is one of the most employed food natural preservatives, belongs to the “Generally Recognized as Safe (GRAS)” category and has been permitted by the United States FDA and by the European Union (E234). Different strategies have been developed to include nisin into NPs for application as food additives with enhanced capacities [174]. Some of these investigations have compared the antimicrobial activity obtained by the addition of free nisin with the activity obtained when incorporating nisin-loaded polymeric NPs. For example, nisin-loaded Ch/Alg NPs (~200 nm of diameter) were evaluated as nanoadditive in pasteurized and raw milks [188]. As control experiments, two more set of samples were analysed, milks (pasteurized and raw) with free nisin and milks without nisin. *S. aureus* bacteria (ATCC 19117) were inoculated in all the samples and the total counts of *S. aureus* were then measured at 24 and 48 h for the pasteurized milk samples and at 0, 6, 10, 14, 18, and 24 h for the raw milk samples. It was shown that the nisin-loaded NPs were able to inhibit the growth of *S. aureus* bacteria more effectively during longer incubation periods than free nisin. Evaluation based on the total counts reveals a similar reduction of *S. aureus* bacteria population in pasteurized milk for free nisin and for nisin-loaded NPs up to 24 h. However, the reduction was more significant for the nisin-loaded NPs at 48 h of incubation. Likewise, a more marked reduction was observed at the longer incubation times (above 14 h) for the raw milk samples containing NPs. This can be explained by the fact that free nisin interacts with ingredients present in the milk and consequently the antimicrobial activity decreases with time, whereas the Ch/Alg NPs protect nisin and prolong its activity. Similar conclusion was obtained when these NPs were added to Feta cheese samples [189].

In another study, nisin has been encapsulated into NPs composed of three biocompatible and food permissible polymers, Ch, Alg and Pluronic F68 with particle size ranging from 130 to 170 nm (as determined by TEM) [190]. The efficiency of the loaded NPs was evaluated in tomato juice and compared with the results obtained with the addition of free nisin and the unloaded NPs. In a control experiment without any additives, the growth of microorganisms (*M. luteus* (MTCC 1809), *P. aeruginosa* (MTCC 424), *S. enterica* (MTCC 1253) and *E. aerogenes* (MTCC 2823)) was observed within 2 days. For unloaded NPs, no growth was observed till 2 months. In the case of free nisin, the growth inhibition was prolonged up to 5 months but seed colour turned black. The best results were obtained with nisin-loaded NPs which exhibited an antimicrobial effect on tomato juice during 6 months. Therefore, it was demonstrated that the antimicrobial activity of nisin was prolonged by its encapsulation in polymeric NPs, although the polymeric NPs containing Ch might contribute to this enhanced activity.

Nanoemulsions containing essential oils have also been developed as nanoadditives to preserve foods from microbial spoilage. Typically, food grade surfactants are employed for this purpose, many of them being polymeric surfactants. For instance, Tween 20 and a starch-derivative have been employed along with other surfactants to encapsulate D-limonene and a mixture of terpenes as antimicrobials [191]. Generally, it was found that the MIC and minimum bactericidal concentration (MBC) values of the nanoencapsulated antimicrobials tested on *E. coli* and Gram-positive *Lactobacillus delbrueckii* bacteria and *Saccharomyces cerevisiae* yeast, were lower than those of free antimicrobials agents. The antibacterial activity of these compounds was also analysed in pear and orange juices inoculated with *L. delbrueckii* (10^3^ CFU/mL) in which a concentration of 5.0 g/L of terpenes was able to totally inactivate the initial microorganisms. The low concentration of antimicrobial required almost did not modify the organoleptic properties of the studied juices. In another example, eugenol-loaded antimicrobial nanoemulsion with droplet diameter of 13 nm was prepared using Tween 80 polymeric surfactant and tested on an orange juice inoculated with *S. aureus* (NCIM 2672) bacteria [192]. Kinetic studies demonstrated that the use of 10% nanoemulsion (0.3% of eugenol in the orange juice) reduces the bacteria population up to 24 h at 4 °C. However, this effect was less marked at 25 °C, with an increase of bacteria population after 6 h. In addition, antimicrobial polymers have been evaluated as emulsifiers in food industry, such as hydrophobically modified ε-polylysine graft copolymers [193].

### 2.3. Environmental Science: Agriculture and Water Purification

Material surfaces exposed to the environment are also susceptible of being easily colonized by microbes present in the soil or transported by the air. In addition, factors such as humidity or temperature favour even more the appearance of biofilms, which are difficult to eradicate. Microbial infections can be ideally prevented by keeping sterile conditions on these surfaces; however, for long-term applications it is necessary to apply extra treatments so that it is possible to maintain such conditions. Traditionally, these methods frequently involve undesired toxic disinfectants such as deoxygenated water, hypochlorite and chemicals that generate reactive oxygen species. The use of specific small biocidal molecules also shows high efficiency in killing microbes; however, their low stability and accumulative toxicity are important issues. Moreover, regular dosages must be applied for all these treatments in order to maintain their activity, which also contribute to the development of antimicrobial resistance.

As mentioned all along this review, the use of polymers as substitutes or accompanying traditional biocides can overcome some of these disadvantages. Polymeric materials, due to their macromolecular properties do not only diminish the riskiness of the low-weight biocides but also improve their stability, usually exhibit long-term activity, are non-volatile, do not permeate through the skin and also offer alternative mechanisms of action helping to fight against antimicrobial resistance.

Regarding chemical functionalities, similar structures to those found in biocides for applications in solution are also present on antimicrobial surfaces. Cationic groups are the most widely employed as it is well known that they interact with the negatively charged bacterial membrane. In particular, functionalities such as *N*-halamines [194,195,196], PEIs [197], phosphonium salts [198,199], quaternary ammonium salts [200,201], and guanidines [202,203,204] are frequently used.

As additional requirements, potential coating materials must provide protection from a broad range of microbial contaminations, maintain its activity for a prolonged period of time and be stable under highly stressing environments. Kim et al. [205] prepared a water resistant adhesive coating based on catechol moieties and quaternized poly(*N*,*N*-dimethylaminoethyl methacrylate) (PDMAEMA). The coating exhibited remarkable bacterial killing efficiency against *S. aureus* (ATCC 25923) and *E. coli* (ATCC 12435) after incubation for 24 h and showed stability up to 60 °C after 60 days in operation. Very recently, these authors [206] have successfully modified PP surfaces. The catechol derivative was quaternized with PPO-*g*-PDMAEMA and the remaining catechol groups were used to attach silver NPs. These surfaces showed unaltered antibacterial activities for 120 h.

Metals, such as copper and silver, are also frequently used as active agents to obtain antimicrobial surfaces. Their strong antimicrobial activities become extremely toxic to bacteria even at exceptionally low concentrations. For this reason, polymer-metal composites have emerged as a highly efficient strategy for a wide range of surface applications. Likewise as NPs [207] and metallic salts, [166,208,209] the number of publications available in literature assessing the antimicrobial properties of these kind of compounds is constantly increasing.

**Figure 4:**
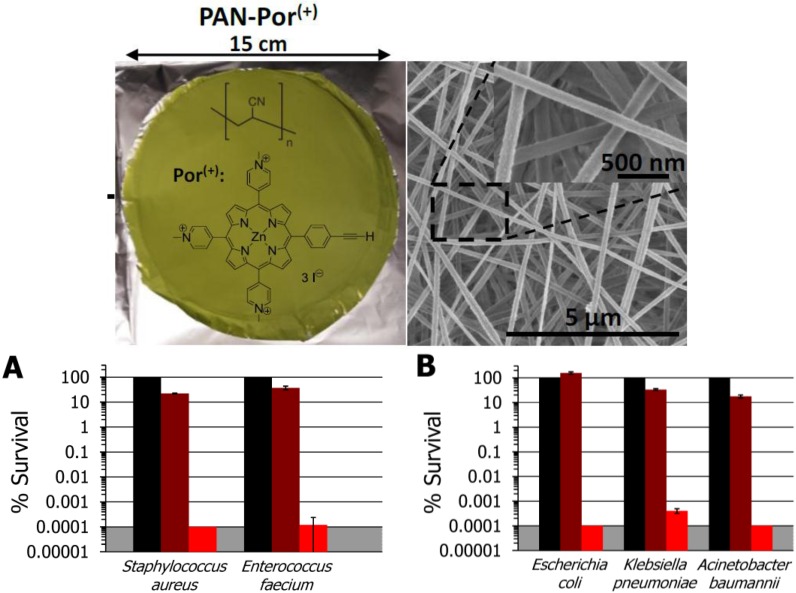
**Upper figures**: Electrospun polyacrylonitrile (PAN)-Por^(+)^ scaffold and scanning electron microscopy images. **Lower figures**: Photodynamic inactivation studies employing PAN-Por^(+)^. (**A**) Gram-positive species: methicillin-susceptible *S. aureus* ATCC-2913 and the vancomycin-resistant *E. faecium* ATCC-2320 strain; (**B**) Gram-negative species: *E. coli* BL21-(Dε3)pLysS, *K. pneumoniae* ATCC-2146, and *A. baumannii* ATCC-19606. For both panels, it is displayed the material-free (cells-only) dark control set to 100% (**black**), as well as the dark control of PAN-Por^(+)^ (**maroon**) and the illuminated PAN-Por^(+)^ (**red**) studies, both as survival rates relative to the material-free (cells-only) dark control. For all bacteria the illumination conditions were as follows: 30 min, 400–700 nm, 65 ± 5 mW/cm^2^ (total fluency of 118 J/cm^2^). Reproduced from [220].

In addition to the use of cationic groups or metallic NPs as antimicrobial agents, photodynamic inactivation is another potential strategy to kill bacteria and unlikely to induce antimicrobial resistance. This mechanism is based on the generation of short-lived singlet oxygen, which is highly oxidative and cytotoxic by inducing the formation of reactive oxygen species inside the cell cytoplasm. These species quickly react with redox enzymes that are crucial for cell survival [210]. Molecules called photosensitizers and photocatalysts are the responsible of forming such species under light irradiation. TiO_2_, ZnO, benzophenones, anthraquinones, and porphyrins are some of the best known compounds with photo-active antimicrobial properties [211]. Wei et al. [212] dispersed Cu and TiO_2_ photocatalytic NPs in an epoxy resin and the activity of the resulting coating was tested against *E. coli* (ATCC 25922) bacteria. Films containing both inorganic compounds showed enhanced antimicrobial activity under sunlight compared to Cu-containing films, making this coating highly suitable for outdoor environmental applications. Porphyrins is one of the approved drugs for photodynamic therapy and some porphyrin-based polymers were reported by Hynek et al. [213]. In order to optimize oxygen production, tridimensionally conjugated microporous polymers were produced by substitution of different porphyrin precursors with appropriate linkers for polymerization via Suzuki-Miyaura cross-coupling reactions. The authors measured the capability of these materials to generate singlet oxygen and it was found that there is a correlation between the amount of singlet oxygen species generated and the 3D environment of the porphyrins. When porphyrins are horizontally stacked, as in the 2D conjugated microporous polymers, or are separated by a short organic-linker, most of the absorbed light energy is dissipated by non-radiative processes. Enlarging the porphyrin separation leads to effective solid photosensitizers that display a significantly higher activity. Porphyrin was also incorporated into PAN electrospun fibres. Figure 4 shows the PAN-Por^(+)^ scaffold and the corresponding scanning electron microscopy image, where the fibres can be clearly observed. Photodynamic inactivation studies performed on Gram-positive and Gram-negative bacteria confirmed the photoactive properties of the material which is inactive in the absence of any irradiation, but is able to destroy the bacteria when illuminated.

Other photosensitive dyes are crystal violet, coumarins, phthalocyanines, rose bengal, eosin, methylene blue or toluidine blue [214,215,216,217,218,219,220]. Although they are less known, they are also capable of effectively inducing formation of reactive oxygen species. The antimicrobial activity against different pathogens has been evaluated on crystal violet alone embedded in poly(dimethylsiloxane) (PDMS) films [221] or combined with ZnO, [222] and methylene blue functionalized silicones [223]. It was demonstrated they possess a good antimicrobial performance under white light. Whitten’s group has extensively worked on this approach [224,225] and they have also proposed a possible mechanism of action for these systems [228]. Although several modes of action have been described in the literature [3,5,110,226,227,228,229,230], this key point is still on debate. However, it seems to be clear that their use reduces the risk of resistance. In addition, the best advantage of the photo-induced antimicrobial systems is their continuous activity under light exposure that makes these materials very interesting for surfaces exposed to the environment.

**Figure 5:**
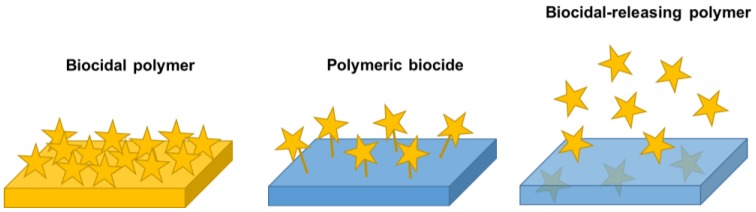
Polymeric surfaces with biocidal activity.

There are many ways to classify polymeric surfaces with biocidal activity but depending on how this function is incorporated into the polymer they can be generally classified as biocidal polymers, polymeric biocides or biocide-releasing polymers [5,210]. Biocidal polymers are those presenting intrinsic antimicrobial activity in their structure; polymeric biocides are those carrying the active molecules covalently linked onto their backbone; and biocide-releasing polymers, in which the polymers act as delivery platforms of small biocides to the environment under diverse conditions (Figure 5). Then, surfaces based on biocidal polymers or polymeric biocides kill microorganism upon contact in contrast to the mechanism of action of the releasing systems. When considering the design of the antimicrobial surface, contact-killing surfaces are preferred compared to releasing surfaces as these possess limited shelf life, are more likely to promote antimicrobial resistance and can cause toxicity to the environment after sustained exposure.

Kumar et al. [231] presented contact-killing surfaces based on nitrogen-rich hyperbanched polyurethanes with intrinsic antimicrobial activity against both bacteria types and some fungi. Alternatively, other authors selected covalent modification of polytetrahydrofurane or polyurethane matrices with azetidinium salts or quaternary ammonium compounds, respectively, to prepare non-leaching coatings [232,233,234].

Nonetheless, a major drawback of these surfaces is that their capacities will fail with time due to damage, contamination or, as the case of the contact-killing surfaces, to the accumulation of death microorganisms. For this reason, biocides that possess the ability of self-renewing are desired. That is, their antimicrobial properties should be auto-recovered without the necessity of changing material or applying new treatments. For example, polymeric film prepared by Dorner et al. [235] consisting of an antimicrobial-biodegradable multi-layer film was able to renew its antimicrobial properties during its decomposition process. Following another concept, Yan et al. [236] designed a coating capable to switch from bactericidal to bacteria repellent surface under wet environment, thus promoting self-cleaning of the surface from dead cells.

#### 2.3.1. Antibiofouling Surfaces

Adhesion-resistant surfaces are a kind of antimicrobial surfaces that without requiring the presence of an antimicrobial by itself, indirectly promotes bacteria removal. Basically, these surfaces repel the adhesion of microbes via different physical repulsion techniques thus avoiding biofouling formation on them. Biofouling is one of the major problems associated to surfaces in contact with the marine environment and its elimination is a tedious and highly cost process. In addition, biofouling present on ship hulls increases frictional drag that directly relates with reduced speeds and an increase in fuel consumption meaning higher costs for shipping companies.

The application of several antifouling coatings based on metallic coatings or organometallic-containing paints has been traditionally the most extended approach. Nevertheless, during last years the appearance of severe environmental regulations and health restrictions demands new eco-friendly technologies to fight against biofouling. In this sense, polymers that present antifouling properties by themselves are one of the most promising alternatives nowadays, although non-toxic biocide-releasing systems are also employed [237,238].

**Figure 6:**
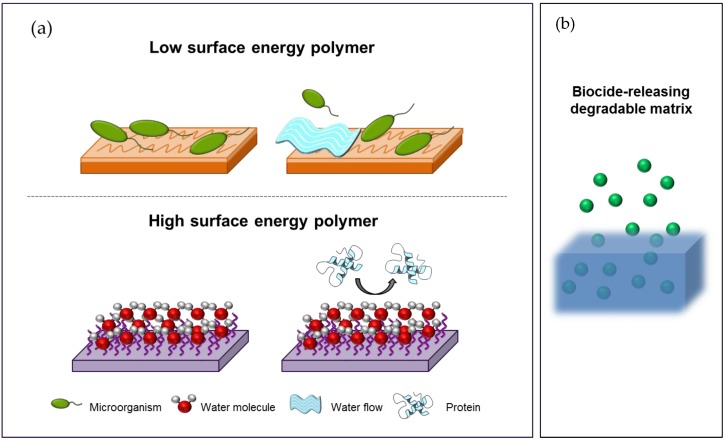
Schematic representation of antibiofouling surfaces. (**a**) Adhesion-resistant surfaces; (**b**) Non-toxic biocide releasing matrices.

It is well known that the intensity of the adhesion of the microorganisms to a particular surface is directly related to the surface energy [239]. Very low surface energies give very weak adhesions that can be reverted by water frictional force (see Figure 6a, top). This is the case of PDMS or fluorinated polymers, with surface energies below 20 mN/m. Oppositely, materials with very high surface energies are also highly hydrophilic and tend to retain a water layer on top of them. Then, interaction of proteins and other biomolecules is impeded as it requires previous rearrangement of water molecules on the surface, which is a thermodynamically unfavourable process (see Figure 6a, bottom). Poly(ethylene glycol)-derivatives belongs to this second group and are probably the most studied antifouling polymers. In this sense, Li et al. [240] prepared low energy slippery surfaces based on hydrophobic porous poly(butyl methacrylate-*co*-ethylene dimethacrylate) copolymer. It was shown that their antifouling properties against *P. aeruginosa* biofilms were highly dependent on the bacteria strain studied, viz. wild type and laboratory strains. On the other hand, Yang et al. [241] obtained multiple antifouling/bactericidal coatings by anchoring active polymeric brushes onto barnacle cement via grafting from and click chemistry techniques. These surfaces were modified with hydrophobic and hydrophilic brushes of poly(poly(ethyleneglycol) methylether methacrylate), poly(2,3,4,5,6-pentafluorostyrene), poly(*N*-hydroxyethyl acrylamide) and poly(2-(methacryloyloxy) ethyltrimethylammonium chloride). Reduction of both adsorption of bovine serum albumin protein and bacteria fouling of *E. coli* (ATCC 14948) and *S. epidermidis* (ATCC 36984) was demonstrated.

Although less frequent, antifouling coatings based on the release of non-toxic biocides from polymeric matrices are also considered. To select the correct matrix, it is necessary to attend at the polymer degradation rates as it will alter the release profile of the active agents and will affect the mechanical properties and shelf life of the film. Low water soluble matrices degrade slowly but protect better from corrosion and oxidation; however, they are not efficient in long-term prevention of biofouling formation. Then, partially water-soluble matrices are preferred as they offer a better control on the biocide release (Figure 6b). For example, a PCL/poly(butylene succinate) blend was employed as carrier for the release of organic antifoulant (4,5-dichloro-2-octyl-isothiazolone) [242]. The antifoulant was released at a constant rate as the blend degrades in marine environment, which can be also modulated by the blend composition. Similarly, copper oxides have also been frequently used as active agent in antibiofouling formulations [243]. However, dissolution of such biocides used in self-polishing coatings leads to their accumulation in marinas, thus resulting toxic to aquatic life. In this case, low-releasing antifouling coatings can be obtained by carrying out the polymerization of copper NPs functionalized with acrylic moieties [244]. In this way, copper NPs became embedded in the polymer backbone limiting its freedom to leach out. Alternatively, Movahedi et al. [245] proposed to make use of the copper naturally present in the seawater to load microparticles of poly(tris[(benzyltriazol) methyl]amine). These particles, which are embedded into a matrix of PMMA, specifically coordinate this element into its structure but in such a way that the copper is still bioavailable for the microorganisms to take it up.

#### 2.3.2. Agriculture

Although polymers were initially used as mere structural materials in the fabrication of greenhouses, nowadays new smart polymeric nanomaterials and delivery systems help the agricultural industry in many different ways. Polymers can enhance the capability of the plants to absorb nutrients, protect the environment from pollutants through filters or combat viruses and other crop pathogens. For example, the presence of parasites that takes away nutrients from the soil results in a decreased land production. This problem has been solved by covering the soil with certain polymeric films able to keep the soil warm during the nights by reflecting the infrared light emitted by the earth. This process ensures decontamination of the soil before seeding and the replacement of highly toxic materials traditionally used such as methyl bromide. Besides, the use of biodegradable polymers for this purpose as those carrying PVA or alginates, [246,247] offers extra advantages as their auto-elimination after use. These materials degrade in eco-friendly sub-molecules saving costs associated with their ‘special’ disposal and reducing environmental pollution derived from its illegal burning.

**Figure 7:**
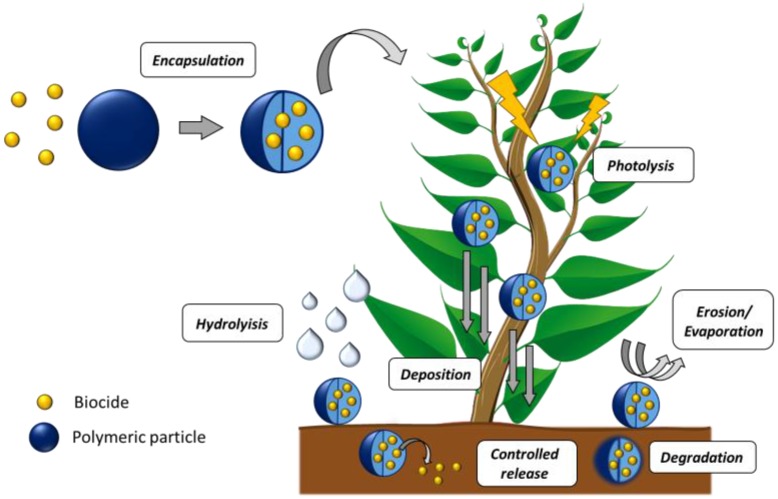
Application of nanotechnology in pesticide delivery.

In particular, biocidal polymers can be employed to increase the efficiency of pesticides and herbicides promoting at the same time the usage of lower doses. This is important as a prolonged exposure to these agrochemicals not only threats operators’ health but also small biocides can easily propagate into the food chain. In addition, due to the environmental conditions that they are exposed to, the majority of the biocidal dose applied is lost and do not reach to complete its action. However, when these molecules are supported on solid polymeric matrices, their toxicity is considerably reduced and their stability under environmental conditions, such as temperature, light or humidity, improved [248]. In addition, encapsulation of biocides can protect from degradation and can modulate their release rates allowing a prolonged delivery over the time, supplying the right quantities of active agent for an efficient action without causing damage to the crop (Figure 7). From this point of view, several authors have described the preparation of polymeric systems based on polyurea resins or biodegradable poly(3-hydroxybutyrate) for the nanoencapsulation of light-sensitive pesticides, fungicides or herbicides as avermeticin [249], phoxim [250] or metribuzin [251], among others [252,253].

**Figure 8:**
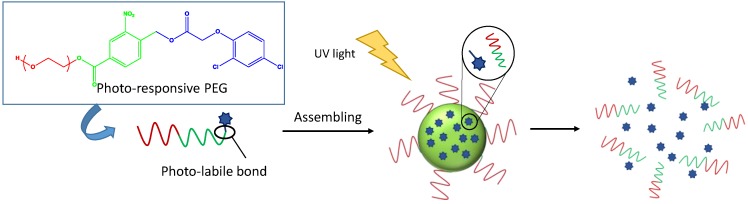
Schematic illustration for the assembly process of a photo-sensitive polymer-agrochemical conjugate and its photocleavage process under light irradiation and subsequent cargo release.

Regarding controlled release of biocides, stimuli-responsive systems, which release their cargo under UV light irradiation, are particularly useful in terms of environmental applications. Tan et al. [254] prepared photo-responsive polymeric particles by creating a dual crosslinked structure. A permanent covalent crosslinking and a reversible CD-based host-guess interaction keep the initial closed form of the particles. However, under UV light, the guess undergoes changes on its conformation disrupting its interactions with the host. As consequence, the structure opens, permitting the release of the loaded content. Similarly, Ding et al. [255] synthetized a self-assembled photo-responsive PEG with the photolabile *o*-nitrobenzyl group and dichlorophenoxyacetic acid grafting (2,4-D),(2,4-D-NBA-PEG) (see Figure 8). The amphiphilic characteristics of this complex allow the system to assemble into nanometric micelles in water protecting the cargo. The release of the active agent takes place in a controlled fashion due to the photolytic cleavage of the link between the polymer and the herbicide under UV light. Most importantly in agriculture, the sunlight, which possesses a low UV intensity, is the source of light and the main controller of that release.

Plastic films are another big giant in the agriculture market. However, and as mentioned before, it is well known that most of plastics used today are non-degradable and their recyclability is difficult and expensive. This is the case of commodity thermoplastics such as PE or PP widely used in agriculture due to their good mechanical and processability properties. Also, abusive use of biocides is generating an important problem of environmental contamination and biocidal-resistance. Thus, the main concern today is to obtain more environmental friendly plastics maintaining at the same time the other requirements. Eco-friendly polymeric biocides eliminate any or both of these issues. For example, biodegradable polymers as PLA and PCL, [256] or some natural polymers such as polysaccharides [257,258] can be used instead of the non-biodegradable polymer matrices as well as natural biocides as some natural oils can be incorporated as active agents [259]. Mallakpour et al. [260] described a polymeric film based on a poly(amino acid) derived from *N*,*N*’-(pyromellitoyl)-*bis*-l-tyrosine dimethyl ester that was biodegradable and biologically active at the same time. In another example lavandin essential oil, a natural biocide, was encapsulated into biodegradable PEG and into *n*-octenyl succinic-modified starch as controlled release platforms [261]. Undoubtedly, combination of a biodegradable polymer and a natural biocide is the most ecological alternative.

#### 2.3.3. Water Purification

The treatment of water to eliminate microbial contamination not only concerns public health. Biofilm and biofouling microbial contaminations can also cause corrosion, souring and plugging of wells and reservoirs, and reduced flow rates. Then, the formation of biofouling is a main concern in the water distribution and filtration technologies and the complete cleaning from inconvenient microbes is still a challenge. The use of chlorine or other water-soluble disinfectants for this purpose, although effective, is associated with problems of residual toxicity. Even if minimal amounts of the substance are used, toxic residues can accumulate in food, in drinking water or in the environment. In addition, chlorine ions react with organic substances present in water yielding to trihalomethane analogs that are suspected of being carcinogenic. For these reasons, their use should be avoided and removal of microorganisms from water with non-soluble polymeric disinfectants turns into the best method for water purification. This means that the use of antimicrobials supported on membranes, fibres or as surface coatings, which kill microorganism by contact instead of by releasing biocidal agents, eliminates the major associated drawbacks. Nevertheless, some water soluble polymeric biocides have been considered for water purification as long as their toxicity is demonstrated to be low enough to suppose a health risk. For example, polyhexamethylene guanidine hydrochloride, a cationic biocide currently used in the treatment of drinking water, was blended with PEG and the leaching of the active to the medium was carefully examined. Quantities of 0.35–0.5 ppm of the biocide were constantly released along 250 L of passing water, which is enough for bacteria removal but still safe for humans [202].

Regarding insoluble disinfectants, the most common ones are mainly crosslinked anion-exchange resins, including quaternary ammonium-type resins, macroporous and macroreticular resins, polyiodide resins, and insoluble polyelectrolytes [262]. Alshehri et al. [263] synthetized a curcumin formaldehyde resin to purify polyphenols present in wastewater, which also showed good activity against several bacterial and fungal strains compared to a standard drug, kanamycin. Also, a set of non-soluble polyacrylonitrile (PAN) films modified with amines of different chain length were prepared by Alamri et al. [264]. The antimicrobial activity was introduced by linking benzaldehyde-derivative moieties onto the amine-functionalized films. Bactericidal efficiency was tested against patient-isolated Gram-positive (*S. aureus*) and Gram-negative (*P. aeruginosa*, *E. coli* and *Salmonella typhi*) bacterial strains, and against some patient-isolated fungi (*Aspergillus flavus*, *A. niger*, *Candida albicans*, *Cryptpcoccus neoformans*). The result showed an increasing efficiency with the amount of phenolic hydroxyl groups into the structure of the polymer. In addition, Bonenfant et al. [265] performed the modification of carboxymethylcellulose and β-CD-based polymers with different quaternary ammonium compounds and these compounds were successfully used in the removal of *E. coli* from wastewater.

Regarding water purification systems, special attention has been placed on *N*-halamines as active agents [194,266]. *N*-halamine and its derivatives have appeared as superior biocides in terms of efficiency, long-term stability, and recharge ability in the treatment of microbial contaminations. These are compounds which possess a nitrogen atom covalently linked to a halogen (X), normally chlorine. Unlike other undesirable chlorine sources, its antimicrobial activity relies on the direct transfer of the oxidative halogen (X^+^) from the *N*-halamine nitrogen to the cell wall of the organism by direct contact followed by oxidation of the lipids in the microbial membrane, rather than dissociation of X^+^ into water followed by diffusion over to a cell. This particular mechanism of action makes these compounds safe to be used in terms of toxicity, as ions do not leach to the surrounding medium to perform its action. In addition, these compounds can be regenerated after exposure of these surfaces to a chlorine source as bleach allowing their reusability during several cycles. As an example, *N*-halamine-modified surfaces did not show loss of integrity after exposure to the equivalent of 300 washing cycles with abrasive chemicals demonstrating the high stability of these compounds. Besides the surface recovered 100% of their activity after 100 chlorination cycles [267]. *N*-halamines used as purification systems can be found in many other examples. Jiang et al. [268] polymerized 2-acrylamido-2-methyl-1-(5-methylhydantoinyl)propane monomer with 3-(trimethoxysilyl)-propyl methacrylate and covalently attached it onto silica gel and sand particles. After chlorination, a contaminated water flow was passed through the particles packed into a column and bacteria population was reduced in 7 logs within 10 s of contact time, demonstrating its high efficiency in water treatment. *N*-bromo-dimethylhydantoin-polystyrene beads were also tested in the removal of *E. coli* (ATCC 15597) and bacteriaphage MS2 (ATCC 15597-B1) from contaminated water according to the NSF-231 Standard Protocol for Testing Microbiological Water Purifiers. Beads were effective in the purification of 550 L and its production was scaled up to kilograms showing potential use as purification systems at industrial scale [269].

An alternative approach to remove microbes from water is the employment of metal-polymer nanocomposites. The small size of the NPs limits their use in some applications that require robust surfaces. Then, metallic NPs have been commonly embedded into polymer matrices forming nanocomposites in order to provide them with the necessary substrate to extend their applications on solid surfaces as those required in water purification [270,271]. Silver NPs were immobilized in a multi-layer film of weak electrolytes, PALA hydrochloride and poly (acrylic acid) (PAA), and a final crosslinking step with glutaraldehyde was applied to reduce the water solubility of the polymer layers thus enhancing the stability of the material for long-term applications. Antimicrobial activity was then evaluated against Gram-positive and Gram-negative bacteria as well as against industrial wastewater where a reduction of 90% of the present coliforms was achieved. In a similar manner, Liu et al. [272] incorporated silver NPs onto a membrane made of PAN following a layer-by-layer approach and subsequent crosslinking. The resulting membrane showed activity against both types of bacteria, positive and negative, and more important the presence of the NPs did not affect membrane performance. This observation is crucial as nanofiltration through membranes is an important section of the water treatment market. In fact, this technique offers some advantages including high water flux, low operation pressure, less energy consumption, and low operation and maintenance costs.

Jewrajka et al. [273] also described the preparation of a silver-polymer nanocomposite for ultrafiltration purposes. The authors synthesized a set of poly(acrylonitrile-*co*-acrylic acid) membranes by mixing PAN/PAN-*co*-PAA/PAN-*co*-PAA-Ag at different ratios and their properties were compared in terms of membrane performance and antimicrobial activity. Membranes containing both copolymer and silver NPs showed better wettability and water flux in addition to good bactericidal properties. Furthermore, the addition of a small amount of PEG as additive in the blend improved the protein antifouling capabilities of the membranes. It is worth to mention that less than 1 wt % of these nanofillers were used to prepare these formulations. This means that good antimicrobial yields can be obtained without necessarily have a massive increase of costs. To overcome the poor water-solubility issue of some biocidal polymers, inorganic particles such as silica or sand can be used as substrates. In this sense, magnetic silica NPs were decorated with polymeric brushes of oligo(ethylene glycol) methacrylate coupled to a natural antimicrobial peptide, Magainin I, and their antibacterial activity was tested against Gram-positive *Listeria ivanovii* [274]. In addition to their antimicrobial properties, these particles also offered the possibility to be easily retained and re-dispersed in aqueous media as many times as desired just by applying an external magnetic field.

#### 2.3.4. Air Purification

As previously mentioned, particles of both biological and non-biological origin can be transported by the air. Bacterial and fungal cells, endospores and spores can travel through this way and infect the environment even when the infection focus is situated far away from it. Then, air filtration is important in order to control such spreading and purify the air especially in susceptible areas such as hospitals or food and pharmaceutical manufacturing industries. Some of the common fungal strains isolated from air filters belong to genera *Aspergillus*, *Penicillium*, *Cladosporium*, *Phoma* and *Mucor*, and regarding bacterial strains *Micrococcus*, *Staphylococcus*, *Pseudomonas* or *Bacillus* have been found among too many others.

The use of antimicrobial polymers-containing filters, as bioaerosol filters, helps to solve this issue [275,276,277]. Natarajan et al. [276] described a PP-based filter modified with polyaniline, which showed high efficiency in the removal of *E. coli*, *B. subtillis* and *S. aureus* strains from contaminated air. Taylor et al. [277] also evaluated the activity of HEPA (High Efficiency Particle Arresting) filters made of polyurethane fibres functionalized with quaternary ammonium antimicrobial groups. These filters were tested against the main bacteria strains commonly responsible for nosocomial infections in hospitals including some multi-resistant species. The successful results against all the strains revealed them as good filters with broad-spectrum bactericidal properties. It is worth to point out the importance of using filters able not only to retain but also to kill the entrapped microorganisms, because it is equally important to reduce the risk of a secondary infection. However, the information is limited and only few examples can be found in literature in the last few years. Then, more research on this field would be necessary given the importance of this matter.

### 2.4. Fabrics

Fabrics or textiles are any cloth made from yarn or fibres by weaving, knitting, felting, etc. As we have shown in the previous sections, the society demands clothes with antimicrobial properties to avoid contamination and/or bad smells during sports/leisure practicing, in addition to those used in hospitals or sanitary services. Likewise, this implies the use of polymers with intrinsic antimicrobial activity, or their chemical or physical modification to incorporate organic or inorganic antimicrobial compounds [4]. Cotton is undoubtedly the polymer most consumed in this context and the most studied. Worley’s group [278,279,280] has extensively applied halamine compounds to enhance the antimicrobial activity of natural, mainly cotton, but also synthetic fabrics, such as PP or PAN. Cotton coatings based on hydantoin diol were also combined with TiO_2_ NPs. Those fabrics showed excellent antimicrobial properties against *S. aureus* (ATCC 6538) and *E. coli* O157:H7 (ATCC 43895) bacteria. Besides, their stability under UV light was enhanced [280]. Cotton fabric was also loaded with graphene oxide by dipping coating method and then immersed in TiCl_3_ aqueous solution acting as both a reducing agent and a precursor to yield a fabric coated with graphene/TiO_2_ nanocomposite. The material was able to self-clean by titania photocatalytic action and to eliminate *S. aureus* (ATCC 25923), *E. coli* (ATCC 25922) bacteria and *C. albicans* (NCPF 3153) fungi [281]. Titania NPs have been also introduced in synthetic fabrics, such as nylon, to obtain protective clothing [282].

Equally, silver NPs were quickly implemented in fabrics [283,284]. Also, silver and ZnO NPs were used to support dyes in cotton and their activity was proved against *S. cerevisiae* yeast [285].

Durability and healing characteristics are other properties hardly pursuit in fabrics. Recently, the synergy between different methodologies was able to create cotton with tuneable colours and durable antibacterial and self-healing superhydrophobic properties. For that, cotton was treated by the solution-dipping method which involved a three-step sequential deposition of branched poly(ethylenimine) (PEI), Ag NPs of tailored colours, and fluorinated-decyl polyhedral oligomeric silsesquioxane (F-POSS) [286].

## 3. Conclusions and Future Development

Here, we have shown some of the most relevant application areas of nanomaterials based on antimicrobial polymers. In general, nanoscience and nanotechnology have helped to create new systems and, indeed new antimicrobial systems with improved properties. It seems that new strategies can be developed to achieve enhanced performance but no doubt, that combinations of existing ones could be effective and promising approaches. We can find different examples in the literature to this respect; for example, LV-loaded NPs with silver core and mesoporous silica shell, which are able to reduce 7.5 and 15 times the MIC values of pure LV against *E. coli* and *K. pneumoniae* bacteria [287]. Moreover, these NPs can reduce the *E. coli* infection in peritoneal cavity of the mice by nearly three orders of magnitude. Another example is the preparation of surfaces with double effect; viz. able to prevent the adhesion of bacteria and having a bactericidal effect.

In addition to the application areas described in this review, there are many other fields not mentioned in which antimicrobial polymeric nanosystems are either important, such as paint formulations, where indoor or outdoor antibacterial and antifungal properties are desired; [288,289] or water hygiene papers [290,291]. Besides, in the nano-era where graphene and related materials are breakthrough we will see important developments in those areas of applications [292,293].

The research on the development of more potent antimicrobial polymeric materials without compromising the human toxicity is increasing and will be enhanced in the upcoming years. In spite of this, researchers should put more efforts in the use of standardized protocols and microorganisms strains in order to have a more accurate and valuable data, which will allow us to go deeper into the mechanism of how microorganisms act and how we should prevent and/or combat them.

## Figures and Tables

**Table 1:** Antimicrobial nanocomposites based on metal and metal oxide nanoparticles (NPs) in food packaging.

Nanoparticles	Polymer	Tested Food	Reference
Ag NPs	LDPE	Barberry	[154]
Ag NPs	EVOH	Chicken, pork, cheese, lettuce, apples, peels, eggshells	[155]
Ag NPs	PS	-	[156]
Ag NPs	Chitosan	-	[157]
Ag-Zeolite	PLA	-	[158]
Ag-Clay	Agar, zein, PCL	-	[159]
TiO_2_	PP	Lettuce	[160]
TiO_2_	EVOH, PP, PCL	-	[161,162,163,164,165,166]
ZnO	LDPE	Orange juice	[167]
ZnO	PVC	Apple	[168]
ZnO, nisin	PLA	Liquid egg	[169]

## Abbreviations

ACP	amorphous calcium phosphate
Alg	alginate
ALM	alveolar like macrophages
AM	ampicillin
Arg	arginine
AZ	azithromycin
Bla	β-lactamase
BSA	bovine serum albumin
CaP	calcium phosphate
CD	cyclodextrin
CFU	Colony-forming unit
Ch	chitosan
CHX	chlorhexidine
CL	clarithromycin
CP	ciprofloxacin
DOTAP	1,2-dioleoyl-3-trimethylammonium-propane chloride salt
DS	dextran sulfate
EVA	ethylene/vinyl acetate copolymer
EVOH	ethylene-vinyl alcohol copolymer
FDA	Food and Drug Administration
F-POSS	fluorinated-decyl polyhedral oligomeric silsesquioxane
Gg	gellan gum
GRAS	Generally Recognized as Safe
GT	gentamicin
HA	hydroxyapatite
HEPA	high efficiency particle arresting
Hy	hyaluronic acid
IFN-γ	interferon-gamma
IL-12	interleukin-12
LCST	lower critical solution temperature
LDPE	low-density polyethylene
LV	levofloxacin
MBC	minimum bactericidal concentration
MIC	minimum inhibitory concentration
MRSA	methicillin-resistant *S. aureus*
MSN	mesoporous silica nanoparticles
MSSA	methicillin susceptible *S. aureus*
MX	moxifloxacin
PNIPAM	poly(*N*-isopropyl acrylamide)
NPs	nanoparticles
NR	norfloxacin
OFX	ofloxacin
PAA	polyacid acrylic
PAANa	poly(sodium acrylate)
PALA	polyallylamine
PAN	polyacrylonitrile
pBA	poly(*n*-butyl acrylate)
PBCA	poly(butyl cyanoacrylate)
PCL	polycaprolactone
PDAEMA	poly(2-aminoethyl methacrylate)
PDMS	poly(dimethylsiloxane)
PDO	polydioxanone
PE	polyethylene
PEG	poly(ethylene glycol)
PEI	polyethyleneimine
PEO	poly(ethylene oxide)
PET	poly(ethylene terephthalate)
PGa	penicillin G amidase
PGA	poly(glycolic acid)
PGCL	poly(glycolide-*co*-caprolactone)
PLA	poly(lactide)
PLGA	poly(lactic-*co*-glycolic acid)
PMMA	poly(methyl methacrylate)
p-(NβGlcEAM)	poly(*N*-2-(β-d-glucosyloxy)ethyl acrylamide)
p(NβGalEAM)	poly(*N*-2-(β-d-galactosyloxy)ethyl acrylamide)
Por	Porphyrins
PPO	poly(propylene oxide)
PP	polypropylene
PS	polystyrene
PU	polyurethane
PVA	poly(vinyl alcohol)
PVP	poly(*N*-vinylpyrrolidone)
RIF	Rifampicin
ROS	reactive oxygen species
TDI	toluene-2,4-diisocyanate
TNF-α	tumour necrosis factor-α
TOB	tobramycin
TPP	tripolyphosphate
VC	vancomycin
